# A health technology assessment of personalized nutrition interventions using the EUnetHTA HTA Core Model

**DOI:** 10.1017/S0266462324000060

**Published:** 2024-03-06

**Authors:** Milanne Maria Johanna Galekop, Josep Maria del Bas, Philip C. Calder, Carin A. Uyl-De Groot, William Ken Redekop

**Affiliations:** 1 Erasmus School of Health, Policy and Management, Erasmus University Rotterdam, Rotterdam, The Netherlands; 2 Eurecat Centre Tecnològic de Catalunya, Biotechnology Area, Reus, Spain; 3 School of Human Development and Health, Faculty of Medicine, University of Southampton, Southampton, UK; 4 NIHR Southampton Biomedical Research Centre, University Hospital Southampton NHS Foundation Trust and University of Southampton, Southampton, UK

**Keywords:** health technology assessment, personalized nutrition, obesity, HTA Core Model, prevention

## Abstract

**Objectives:**

Poor nutrition links to chronic diseases, emphasizing the need for optimized diets. The EU-funded project PREVENTOMICS, introduced personalized nutrition to address this. This study aims to perform a health technology assessment (HTA) comparing personalized nutrition interventions developed through this project, with non-personalized nutrition interventions (control) for people with normal weight, overweight, or obesity. The goal is to support decisions about further development and implementation of personalized nutrition.

**Methods:**

The PREVENTOMICS interventions were evaluated using the European Network for HTA Core Model, which includes a methodological framework that encompasses different domains for value assessment. Information was gathered via [1] different statistical analyses and modeling studies, [2] questions asked of project partners and, [3] other (un)published materials.

**Results:**

Clinical trials of PREVENTOMICS interventions demonstrated different body mass index changes compared to control; differences ranged from −0.80 to 0.20 kg/m^2^. Long-term outcome predictions showed generally improved health outcomes for the interventions; some appeared cost-effective (e.g., interventions in UK). Ethical concerns around health inequality and the lack of specific legal regulations for personalized nutrition interventions were identified. Choice modeling studies indicated openness to personalized nutrition interventions; decisions were primarily affected by intervention’s price.

**Conclusions:**

PREVENTOMICS clinical trials have shown promising effectiveness with no major safety concerns, although uncertainties about effectiveness exist due to small samples (n=60–264) and short follow-ups (10–16 weeks). Larger, longer trials are needed for robust evidence before implementation could be considered. Among other considerations, developers should explore financing options and collaborate with policymakers to prevent exclusion of specific groups due to information shortages.

## Introduction

Poor nutrition is a cause of chronic diseases such as ischemic heart disease (IHD), stroke, obesity, and type 2 diabetes (1;2). In 2019, dietary risk factors contributed globally to approximately 7.94 million deaths and 188 million disability-adjusted life years among people aged 25 years and older (3). Moreover, dietary factors account for approximately 18.2 percent of the costs associated with IHD, stroke, and type 2 diabetes in the United States (2). Personalized nutrition has emerged as a promising field to address the limitations of current diet interventions and slow down the chronic disease pandemic (1). Since each individual has different nutrient needs and responses to diets, insights into these individual needs and responses can be leveraged to prevent, manage, and treat diseases and to improve health (4). Personalized nutrition has been defined by Ordovas et al. (5) as an approach that utilizes individual characteristics to provide targeted nutritional advice, products, or services. To develop such advice, products, or services, clinical assessments, biomarkers of physiological function and pathological processes, genetic information, and other available data derived from advanced technologies are needed (1).

While information on lifestyle and personal goals is commonly used to formulate personalized nutrition advice, the same is not true for advanced technologies such as those involving metabolomics and genotypic data, despite their potential to improve health outcomes (6;7). One project that explored the potential of advanced technologies in people with normal weight, overweight, and obesity is PREVENTOMICS, a recently completed European Horizon 2020 project (8), which investigated the potential of omics (especially metabolomics) as an input for personalized nutrition advice (9). By combining phenotypic characterization at the metabolomic level with a person’s genotype, lifestyle, health status, preferences, and physiological status, a novel platform was developed and integrated into third-party applications. This integration resulted in three PREVENTOMICS interventions (9), which included the following: [1] integration of the platform for personalized food delivery, [2] integration of the platform at the retailer level for personalized recommendations when shopping, and [3] integration of the platform with a software to support healthcare professionals with formulating personalized dietary plans for consumers (10).

Decisions regarding the implementation of new approaches in healthcare, such as PREVENTOMICS, are rarely simple (11). Growing pressure on healthcare budgets has resulted in increased scrutiny of the overall value of new health technologies and programs (12). In this context, the importance of conducting a health technology assessment (HTA) is emphasized. HTA is a “multidisciplinary process that uses explicit methods to determine the value of a health technology at different points in its lifecycle” (13). “Value” includes different dimensions, such as clinical effectiveness, safety, costs, and ethical and legal issues. HTA promotes transparency and accountability in government performance, and it can also help developers of new technologies in understanding how their technology will be assessed (i.e., early HTA); by conducting such an “early HTA”, the time and financing required for their product to gain market entry or get reimbursed can potentially be reduced (14;15).

Previous HTAs have often assessed only the costs, health effects, and cost-effectiveness of nutrition interventions and have not systematically examined a wider range of possible issues relating to health care and society (16). To overcome the variance in the extent and scope of HTA, and the differences in reporting of the results, the European Network for HTA (EUnetHTA) developed the HTA Core Model (17). Conducting an (early) HTA with the HTA Core Model offers advantages such as the identification of key assessment components of interventions, the provision of a structured analysis of (early) scientific evidence, and the highlight of existing gaps from which the recommendations for subsequent decision-making steps can be formulated (18). Despite these benefits, only a limited number of studies utilizing the HTA Core Model for HTA have been published in scientific journals (19–21), and none of them were conducted in the nutrition field. As we believe that assessing the PREVENTOMICS interventions with the HTA Core Model in the premarket phase can help to inform further development and potential implementation decisions, this study aimed to compare these interventions with non-personalized nutrition interventions for people with normal weight, overweight and obesity, on all of the domains found in the HTA Core Model.

## Materials and methods

### General information regarding the HTA Core Model

The PREVENTOMICS interventions were evaluated using the HTA Core model developed by EUnetHTA, which has nine domains covering all aspects of an HTA (see Table 1) (22). This model was chosen because of its methodological framework for producing and sharing HTA information (22). Alternative frameworks were evaluated but not selected for various reasons. For example, the ISPOR Value Flower, which offers a broader perspective on factors contributing to value in healthcare, was not chosen because it predominantly centers on the concept and measurement of value rather than on the process and execution of HTA (23). The methodological framework of the HTA Core Model includes three components: [1] an HTA ontology including standardized questions (i.e., assessment elements) organized within a framework featuring nine domains that encompass all aspects that may be relevant for HTA and thereby value assessment, [2] methodological guidance, and [3] a common reporting structure. We used the first two components of the framework wherever possible. We did not use the common reporting structure and instead provided a summary of the relevant information per domain related to the PREVENTOMICS interventions, which gives a streamlined and accessible documentation of essential information. We believe that this is sufficient for stakeholders who are interested in further development or in taking (decision-making) steps regarding the implementation of the interventions.

**Table 1.** Different domains of the HTA Core Model, including the related methodology and sources used to address the domain

Domains of HTA		Domain description as summarized in this study	Deliverable(s) (D) used^a^	Other sources
		This domain summarizes (25):		
1	Health problem and current use of technology	Target conditions + societal and individual burden of these conditions Study populations Current management. Knowledge is crucial for contextualizing and understanding outcomes observed in the other domains.	D1.2 (“Consumers Report”) (published)^b^ D7.5 (“Final plan for the Use and Dissemination of Results-PUDR”)	Ghelanie et al. (36) Keijer et al. (9) OECD (35) PREVENTOMICS website (8)
2	Description and technical characteristics of technology	Technical characteristics (e.g., users of the technologies) Materials and equipment Staff needed (and its training) The regulatory status (i.e., the reimbursement policies of the technologies) Since even minor variations in technologies may result in different outcomes, this domain is of great importance.	D4.1 (“PREVENTOMICS platform design”) D5.3 (“Report on the outcome of each intervention study”) D7.5 (“Final plan for the Use and Dissemination of Results-PUDR”) D9.1 (“Requirement N°1-Humans Interventional studies“)	Aldubayan et al. (26) Aldubayan et al. (37) Bothos (10) Bush et al. (1) Calder (40) Del Bas (38) Gerke et al. (43) Keijer et al. (9) Malczewska-Malec (39) Poley (42) PREVENTOMICS website (8) Van Berlo (41)
3	Safety	Safety issues (unwanted or harmful consequences) that are important to participants Or otherwise likely to be important in guiding decisions of stakeholders This could be related to occupational, and environmental safety.	-	Klingler et al. (45) NIDDK (44) Via questions asked via email to partners of the PREVENTOMICS project, who are experts in this field.
4	Clinical effectiveness	Health benefits including: Mortality Morbidity Quality of life	D5.3 (“Report on the outcome of each intervention study”) D5.4 (“Overall performance of PREVENTOMICS service”) D6.4 (“Cost-effectiveness analyses results”)	Aldubayan et al. (26) Clamp and Baker (46) Galekop et al. (27) Galekop et al. (28) Galekop et al. (29) Hoogendoorn et al. (32) Malczewsk-Malec et al. (47) Rabassa et al. (48)
5	Costs and economic evaluation	Costs Health outcomes Economic efficiency information. Crucial given rising healthcare costs and limited healthcare budgets	D5.3 (“Report on the outcome of each intervention study”) D6.4 (“Cost-effectiveness analyses results”)	Galekop et al. (27) Galekop et al. (28) Galekop et al. (29) Hoogendoorn et al. (32)
6	Ethical aspects	Social and moral norms and values, such as: Benefit–harm balance Autonomy Respect for persons Justice and equity Legislation Ethical consequences of the HTA Important to assess since moral values and norms, being the foundation of social life, significantly influence the way in which PREVENTOMICS interventions can be used in practice.	D1.2 (“Consumers” report”) (published)^b^ D7.2 (“Data management plan”)	Mathers (49) Via questions asked via email to partners of the PREVENTOMICS project, who are experts in this field.
7	Organizational aspects	Mobilizing and organizing resources, including human skills and material artifacts, needed for implementation. Done by focusing on: Health care system structure and delivery process Management Culture Implementation challenges and barriers	-	Via questions asked via email to partners of the PREVENTOMICS project, who are experts in this field.
8	Patients and social aspects	Issues for: Individuals^c^ Caregivers Social groups Understanding individual perspectives is crucial as they provide unique insights into experiences, attitudes, preferences, values, and expectations.	D1.2 (“Consumers” report”) (published)^b^ D5.3 (“Report on the outcome of each intervention study”) D6.4 (“Cost-effectiveness analyses results”)	Farrell et al. (50) Harris et al. (51) Galekop et al. (34)
9	Legal aspects	Individual’s autonomy Privacy Health equality Rules and regulations protecting participant rights and societal interest.	D6.1 (“Ethical framework”) D6.2 (“Regulatory framework”) D7.2 (“Data management plan”) D7.4 (“PUDR”) D7.5 (“Final plan for the Use and Dissemination of Results-PUDR”)	Ahlgren et al. (52) European Commission (54) Rottger-Wirtz & De Boer (53)

a All results were part of D6.5 (“Health Technology Assessment”).

b Published online: https://preventomics.eu/deliverables/#1593502709004-84c73ce5-2fe4.

c In this regard, “patient” and “individual” denotes those receiving a technology. This study focused on people without chronic diseases, and therefore the term “Individual” (or “participant”) was used in this HTA.

D, Deliverable; HTA, Health Technology Assessment; PREVENTOMICS, Empowering consumers to PREVENT diet-related diseases through OMICS sciences.

### Domain specific methods

Table 1 gives an overview of all domains, the description of the domains, and the different sources used to gather information. A summary of domain-specific methods is given below. In general, information for the different domains was gathered via [1] different statistical analyses (i.e., analyses of health outcomes and questionnaires) and modeling studies (i.e., cost-effectiveness modeling and choice modeling); [2] questions asked via email to partners of the PREVENTOMICS project, who are experts in this field; or [3] other (un)published materials. Published materials included literature published in scientific journals, PREVENTOMICS blog posts, and presentations. Unpublished materials included project deliverables (D). These deliverables are also known as supplementary outcomes (such as information, specialized reports, or brochures) that were required to be generated at a specific time throughout the project (24). All published materials related to the PREVENTOMICS project can be accessed on the website (8), and information about the referenced deliverables is provided in Supplementary Table S1.

In most domains, (un)published materials were used as input, as well as the questions that were asked of the project partners (see Table 1). Additionally, clinical trial data were used as input for the “clinical effectiveness” and “cost and economic evaluation” domains and were analyzed using statistical methods (see footnote Table 3 for more details), with some results extrapolated over a lifetime. Although some of these results were already published elsewhere (26–29), we provided a summary of the trial-based effectiveness on dietary intake (i.e., Mediterranean Diet Adherence Score), anthropometrics (i.e., body fat, waist circumference, and body mass index (BMI)) and QoL (assessed with the EQ-5D-5L and the Obesity and Weight Loss Quality of Life (OWLQOL)) (30;31).

**Figure 1.**
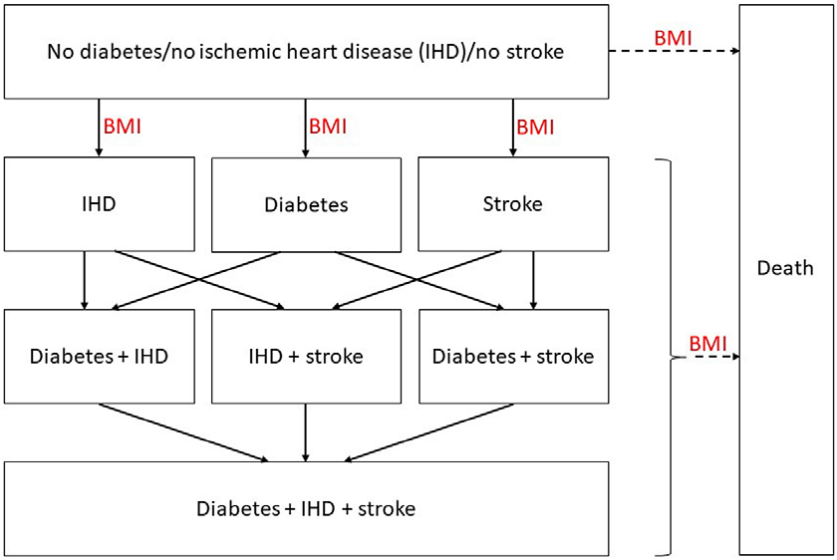
Structure of the Markov model for obesity as presented by Hoogendoorn et al. (32). BMI, body mass index; IHD, ischemic heart disease.

The Markov obesity model with a 1-year cycle length was used to analyze data over a lifetime horizon and had different health states: diabetes, IHD, stroke, and death (see Figure 1 for the model structure) (32). The model simulated the disease occurrence for an obese cohort based on various inputs (e.g., population demographics and trial-based effectiveness on BMI). The effectiveness measure was quality-adjusted life years (QALYs) and the cost-effectiveness was expressed in the incremental cost-utility ratio. More details about the model and inputs can be found elsewhere (32). Detailed lifetime results were published elsewhere (27–29) and summarized in this study.

Input for the “patients and social aspects” domain was supplemented with a validated diet satisfaction questionnaire (DSat-28 (© Laboratory for the Study of Human Ingestive Behavior, The Pennsylvania State University)), that assesses satisfaction with weight-management diets (33). The DSat-28 consists of 28 items with five response options ranging from “disagree strongly” to “agree strongly.” The total score was calculated by averaging the summed score; higher scores indicate greater diet satisfaction. Additionally, preferences regarding personalized nutrition interventions were obtained from results from two published discrete choice experiments (DCEs) (34), that assessed preferences about [1] personalized nutrition advice and [2] personalized meals. More information about the methodology of these DCEs can be found elsewhere (34).

## Results

### Health problem and current use of technology

The PREVENTOMICS interventions were used in four countries (Denmark, the United Kingdom (UK), Poland, and Spain) targeting overweight and obese populations (8). Spain also included individuals with normal weight (see Supplementary Table S2 for obesity classification by BMI). All interventions aimed to prevent diet-related diseases and improve health (8). More details can be found in Table 2.

**Table 2.** Details on the PREVENTOMICS interventions, including information on the different intervention arms, study population, and target condition

Intervention				Study population	Target condition
Country	General information	Intervention group(s)	Control group		
Denmark (37)	Platform used in the elaboration and delivery of personalized food. Integrated with the SimpleFeast app. Trial period: 10-week randomized trial. Meals: vegetarian meals (breakfast and dinner) for 6 days. For the seventh day (Saturday) and for lunches, people needed to prepare meals themselves. They were allowed to eat nonvegetarian food, but were encouraged to refer to the recipe recommendations presented through the SimpleFeast App.	Personalized dietary plan (PP) Personalized easy-to-prepare meal boxes: based on metabolome and genetic analyses, participants were clustered into different groups and receiving group-specific meals. + functional ingredients matching the cluster were added to meals (26) + behavior change program^a^	General dietary plan General easy-to-prepare meal boxes. + behavior change program* (however, Do’s were not personalized and were more informational messages).	Adults aged 18–65 years, with a BMI of 27–40 kg/m^2^ and elevated waist circumference (men >94 cm; women >80 cm). No chronic diseases such as diabetes, heart diseases, or cancer.	Improved diets result in a greater reduction in excess body fat and weight, as well as benefits in overall health in people with overweight and obesity. This could prevent diet-related diseases such as cardiovascular diseases, diabetes, several cancers, and stroke (67).
Spain (38)	Platform used at shop level. Integrated with the ALDI app (i.e., microsite). Trial period: 21-week parallel, randomized, placebo-controlled trial, and single-blind intervention trial. Division into study arms: Based on participants metabolome (urine, plasma, and serum samples) and saliva analysis of different single nucleotide polymorphisms, participants were randomized in a cluster, and divided into one of the three study arms.	Personalized nutrition (PN) Personalized recommendations through the ALDI catalogue. Personalized dietary plan (PP) PN + behavior change program^a^	General dietary plan Recommendations through the ALDI catalogue.	Adults aged 18–65, with a BMI of 18.5–35 kg/m^2^ (general population including those living with obesity), without any chronic disease with clinical manifestation.	Improved dietary habits, measured through the adherence to the Mediterranean diet for people with- and without overweight and obesity, could lead to prevention of different diet-related diseases. This prevention could be [1] via overweight/obesity: reducing weight could prevent high blood pressure, elevated blood lipids, prediabetes, and thereby prevent diet-related diseases (70), or [2] not via overweight/obesity: reduction in high blood pressure because of better nutrition (improvements in Mediterranean diets) might lead to prevention of diseases (71).
Poland/UK (39,40)	Platform used through an upgraded ICT-based software for professionals. Integrated with the MetaDieta app. Trial period: 4-month single-blind randomized, placebo-controlled trials. Division into study arms: Based on participants classical biomarkers (urine, plasma, and serum samples) and saliva analysis (genetic polymorphisms) participants were allocated in a cluster and divided into one of the three study arms.	Personalized nutrition (PN) Personalized diet created by a dietician via a MetaDieta software. Participants themselves could also use the MetaDieta mobile app to support dietary compliance, monitor intake, and contact the dietician. Personalized dietary plan (PP) PN + behavior change program^a^	General dietary plan Dietary plans were based on general healthy eating guidelines.	Adults aged 18–65 years, with a BMI of 25–40 kg/m^2^ and elevated waist circumference (men >94 cm; women >80 cm). People without any chronic diseases or treated with drugs could be included; however, people with hypertension and taking antihypertensive drugs (metabolically neutral) could be included.	The aim of the trial was to reduce weight and waist circumference by improving diets and to get favorable changes in metabolic profile. In turn, this might result in a reduction of abdominal obesity and the related diseases (67).

a Behavioral change program: delivered via ONMI (https://www.onmi.design/preventomics). Participants received two to three Do’s (behavioral prompts) per week. In nature, participants were prompt to take a specific action. The Do’s in the PP group were based on participants’ reports from the behavioral questionnaire at baseline and inputs from nutritional recommendations.

ALDI, supermarket; BMI, body mass index; cm, centimeter; ICT, information, and communication technology; kg, kilogram; m, meter.

The burden of obesity is high; in 2016, over half of the population in OECD countries was overweight and nearly one in four had obesity (35). Poor diet significantly contributes to this obesity epidemic, with almost half of the population not meeting healthy diet guidelines and international standards. Overweight and related co-morbidities reduce average life expectancy in OECD countries by 2.7 years on average (35). Moreover, overweight and obesity result in an economic burden due to increased healthcare costs and reduced productivity. Over the next 30 years, OECD countries are projected to spend an average of 8.4 percent of their health budget on overweight-related problems, leading to a 3.3 percent reduction in gross domestic product due to obesity (35).

Although countries have implemented policies to tackle overweight and obesity, their success has been limited (35). Improvements in specific strategies such as mobile apps to promote healthier lifestyles could potentially tackle overweight and obesity. One study (D1.2 (“Consumers Report”)) and the literature (36) found that many mobile apps for this purpose already exist. However, as far as we know, PREVENTOMICS uses a unique approach by applying new technologies (see “description and technical characteristics of the technology” domain) (9).

### Description and technical characteristics of the technology

The PREVENTOMICS interventions assessed in this HTA involved the use of a platform in different ways. In general, the platform used relevant algorithms and analytics services to analyze user data (genetic, biological, nutritional, psychological) and stored it for providing personalized nutrition recommendations (9). These recommendations were transmitted through three different dietary apps: SimpleFeast, ALDI, and MetaDieta.

In more detail, the first PREVENTOMICS intervention integrated the platform with the SimpleFeast app for personalized meal delivery in Denmark (10;26;37). The second intervention integrated the platform at the retailer level with an ALDI supermarket app in Spain (developed ad hoc), which enabled customers to read personalized food product recommendations while grocery shopping (10;38). The third intervention integrated the platform with the MetaDieta app, designed for use by dieticians and study participants in the UK and Poland (10;39;40). Dieticians used this app to prepare diet plans and share them with the participants. Moreover, all interventions included a behavioral change program (41) (see Table 2 for additional intervention details, Supplementary Figure S1 for the PREVENTOMICS user journey, Supplementary Figures S2a–d for the study designs, and Supplementary Table S3 for required training and tools).

Reimbursement policies for nutrition-related technologies vary both across and within countries. Generally, nutrition interventions or related areas such as digital health tools are not reimbursed (1;42). However, recent initiatives, such as the introduction of the Digital Healthcare Act (Digitale-Versorgung-Gesetz) in Germany, aim to improve healthcare through digitalization and innovation by reimbursing tools such as obesity apps (43) (see Supplementary material S1, for example, of reimbursement policies for different areas related to the PREVENTOMICS interventions in different countries).

### Safety

PREVENTOMICS interventions are generally safe for individuals; no specific safety risks are related to the use of digital tools (a major component of the interventions). However, other activities related to the interventions may have safety hazards. For example, drawing blood (one to two times per year) may cause minor bruising at the puncture site. Moreover, there is a risk of contamination due to improper needle management. To address these concerns, alternatives such as skin monitors for blood glucose measurement (44) or finger pricks (for small blood volumes) (45) can be used. In addition, there is a theoretical possibility that participants could receive the wrong type of personalized nutrition. However, manual checks minimize this risk. Moreover, since all dietary plans are based on the Mediterranean diet, recognized as a healthy diet, any potential error would have limited impact on health outcomes. The interventions do not pose risks to environmental or occupational safety.

## Clinical effectiveness

To summarize the effectiveness of the PREVENTOMICS interventions, both short-term effectiveness (trial-based effectiveness) and long-term effectiveness (modeling trial-based effectiveness over lifetime) were studied (see Table 3) and varied by intervention and country. In both intervention groups (PP and PN: see Table 2 for description) and the control, we observed short-term changes in health outcomes, including shifts in BMI and utilities (i.e., quality of life score) from baseline to follow-up. These shifts were generally associated with improved health (i.e., decreased BMI and improved EQ-5D-5L utilities); BMI change ranged from −1.31 kg/m^2^ (PP group, UK) to 0.08 kg/m^2^ (control, Spain) and utility change ranged from −0.02 (control, Denmark and UK) to 0.06 (PN, UK). Additionally, these changes from baseline to follow-up in PP and PN groups were compared with those in the control group, providing estimates of the difference in effectiveness between interventions and control, accompanied with 95 percent confidence intervals. The highest (statistically significant) effect on BMI was measured when PN was compared with control in Spain (−0.53 kg/m^2^) and in utilities when PP was compared with control in Denmark (0.04). Notably, we observed contrasting effectiveness results in BMI in Poland when PN was compared with control; BMI in the control group decreased more than in the PN group, resulting in a +0.20 kg/m^2^ difference. Analysis of the OWLQOL indicated significant increases in QoL for all PP and PN interventions compared to baseline (e.g., PP in Denmark: +3.85 (SE: 1.67)). However, statistically significant differences in OWLQOL between interventions were generally not observed in most countries, except for PN versus control in Poland.

**Table 3.** Trial and model outcomes related to (discounted) effects, costs, and cost-effectiveness

		PP	PN	Control	Difference PP–control	Difference PN–control
Effects trial period (baseline vs. follow-up)^a^						
Denmark	BMI, in kg/m^2^ (mean, SE, or CI)	−1.05 (0.17)^b^	–	−0.98 (0.15)^b^	−0.07 (−0.51, 0.38)	–
	Body fat, % (mean, SE, or CI)	−1.0 (0.2)^b^	–	−0.9 (0.2)^b^	−0.1 (−0.7, 0.5)	–
	EQ-5D-5L utilities^f^, (mean, SE, or CI)	0.02 (0.01)	–	−0.02 (0.01)	0.04 (0.00, 0.07)^c^	–
	OWLQOL (mean, SE, or CI)	3.85 (1.67)^d^	–	2.58 (1.56)	1.27 (−3.20, 5.75)	–
	DSAT-28^g^, (mean, SE, or CI)	0.24 (0.05)^b^	–	0.17 (0.05)^b^	0.07 (−0.08, 0.27)	–
Spain	BMI, in kg/m^2^ (mean, SE, or CI)	−0.38 (0.13)^b^	−0.45 (0.14)^b^	0.08 (0.15)	−0.46 (−0.85, −0.07)^c^	−0.53 (−0.94, −0.13)^c^
	MEDAS, (mean, SE, or CI)	2.80 (0.25)^b^	2.72 (0.27)^b^	3.01 (0.30)^b^	−0.22 (−1.00, 0.55)	−0.29 (−1.08, 0.50)
	EQ-5D-5L utilities^f^, (mean, SE, or CI)	−0.00 (0.01)	0.01 (0.01)	0.01 (0.01)	−0.01 (−0.04, 0.01)	0.00 (−0.02, 0.03)
	DSat-28^g^, (mean, SE, or CI)	0.13 (0.06)^d^	0.17 (0.07)^d^	0.25 (0.07)^b^	−0.12 (−0.30, 0.06)	−0.09 (−0.27, 0.10)
Poland	BMI, in kg/m^2^ (mean, SE, or CI)	−1.03 (0.23)^b^	−0.63 (0.22)^b^	−0.82 (0.24)^b^	−0.20 (−0.86, 0.45)	0.20 (−0.45, 0.85)
	Waist circumference, cm (mean, SE, or CI)	−4.34 (0.77)^b^	−4.60 (0.74)^b^	−3.65 (0.80)^b^	−0.69 (−2.87, 1.48)	−0.95 (−3.09, 1.19)
	EQ-5D-5L utilities^f^, (mean, SE, or CI)	−0.00 (0.01)	0.01 (0.01)	0.01 (0.01)	−0.01 (−0.04, 0.02)	0.00 (−0.03, 0.03)
	OWLQOL (mean, SE, or CI)	7.75 (1.99)^b^	12.76 (1.91)^b^	5.91 (2.00)^b^	1.84 (−3.68, 7.37)	6.85 (1.43, 12.27)^c^
	DSat-28^g^, (mean, SE, or CI)	−0.03 (0.06)	0.16 (0.06)^b^	0.13 (0.06)^d^	−0.16 (−0.32, −0.00)^c^	0.03 (−0.13, 0.18)
UK	BMI, in kg/m^2^ (mean, SE)	−1.31 (0.27)^b^	−0.84 (0.27)^b^	−0.51 (0.31)	−0.80 (−1.60, 0.00)	−0.33 (−1.14, 0.48)
	Waist circumference, cm (mean, SE, or CI)	−8.80 (0.91)^b^	−6.63 (0.93)^b^	−1.6 (1.05)	−7.20 (−9.91, −4.48)^e^	−5.03 (−7.78, −2.28)^e^
	EQ-5D-5L utilities^f^, (mean, SE, or CI)	0.01 (0.03)	0.06 (0.02)^b^	−0.02 (0.04)	0.03 (−0.07, 0.13)	0.08 (−0.02, 0.17)
	OWLQOL (mean, SE, or CI)	22.4 (4.49)^b^	11.63 (4.60)^d^	12.4 (5.18)^d^	10 (−3.44, 23.44)	−0.77 (−1.4.36, 12.82)
	DSat-28^g^, (mean, SE, or CI)	0.41 (0.12)^b^	0.32 (0.13)^d^	0.36 (0.14)^d^	0.05 (−0.32, 0.41)	−0.04 (−0.41, 0.33)
Effects lifetime period^h^						
Denmark	Life years (base case)^i^	17.766	–	17.763	0.003	–
	Life years with diabetes (base case)^i^	2.769	–	2.781	−0.012	–
	Life years (scenario)^j^	17.784	–	17.763	0.021	–
	Life years with diabetes (scenario)^j^	2.698	–	2.781	−0.082	–
	Cum. Incident cases IHD/1000 (base case)^i^	279.448	–	279.788	−0.34	–
	Cum. Incident cases stroke/1000 (base case)^i^	315.719	–	316.094	−0.375	–
	Cum. Incident cases IHD/1000 (scenario)^j^	277.332	–	279.788	−2.455	–
	Cum. Incident cases stroke/1000 (scenario)^j^	313.381	–	316.094	−2.713	–
	QALYs (base case)^i^	15.117	–	15.106	0.011	–
	QALYs (scenario)^j^	15.139	–	15.106	0.033	–
Spain	Life years (base case)^i^	23.326	23.326	23.324	0.002	0.002
	Life years with diabetes (base case)^i^	1.38	1.374	1.416	−0.037	−0.042
	Life years (scenario)^j^	23.327	23.327	23.324	0.003	0.003
	Life years with diabetes (scenario)^j^	1.35	1.343	1.416	−0.067	−0.074
	Cum. Incident cases IHD/1000 (base case)^i^	148.732	148.537	150.016	−1.284	−1.479
	Cum. Incident cases stroke/1000 (base case)^i^	230.06	229.816	231.669	−1.609	−1.854
	Cum. Incident cases IHD/1000 (scenario)^j^	147.649	147.4	150.016	−2.367	−2.616
	Cum. Incident cases stroke/1000 (scenario)^j^	228.695	228.38	231.669	−2.974	−3.289
	QALYs (base case)^i^	20.158	20.162	20.156	0.002	0.006
	QALYs (scenario)^j^	20.162	20.166	20.156	0.006	0.01
Poland	Life years (base case)^i^	19.385	19.36	19.372	0.013	−0.013
	Life years with diabetes (base case)^i^	2.865	2.952	2.908	−0.043	0.044
	Life years (scenario)^j^	19.425	19.4	19.372	0.053	0.028
	Life years with diabetes (scenario)^j^	2.73	2.813	2.908	−0.179	−0.096
	Cum. Incident cases IHD/1000 (base case)^i^	326.253	328.0997	327.171	−0.919	0.926
	Cum. Incident cases stroke/1000 (base case)^i^	409.089	412.369	410.721	−1.632	1.648
	Cum. Incident cases IHD/1000 (scenario)^j^	323.266	325.113	327.171	−3.905	−2.058
	Cum. Incident cases stroke/1000 (scenario)^j^	403.809	407.07	410.721	−6.913	−3.651
	QALYs (base case)^i^	16.536	16.51	16.525	0.011	−0.015
	QALYs (scenario)^j^	16.585	16.557	16.525	0.057	0.032
UK	Life years (base case)^i^	19.796	19.776	19.762	0.034	0.014
	Life years with diabetes (base case)^i^	1.552	1.596	1.627	−0.075	−0.031
	Life years (scenario)^j^	19.828	19.810	19.762	0.066	0.048
	Life years with diabetes (scenario)^j^	1.483	1.522	1.627	−0.144	−0.105
	Cum. Incident cases IHD/1000 (base case)^i^	341.617	343.553	344.921	−3.304	−1.368
	Cum. Incident cases stroke/1000 (base case)^i^	340.409	342.263	343.577	−3.168	−1.314
	Cum. Incident cases IHD/1000 (scenario)^j^	338.354	340.225	344.921	−6.567	−4.696
	Cum. Incident cases stroke/1000 (scenario)^j^	337.294	339.079	343.577	−6.283	−4.498
	QALYs (base case)^i^	16.023	16.019	15.979	0.044	0.040
	QALYs (scenario)^j^	16.056	16.053	15.979	0.077	0.074
Costs trial period^k^						
Denmark	Total costs, 2020 € (DKK)	7,402 (55,277)	–	5,653 (42,215)	1,749 (13,062)	–
Spain	Total costs, 2020 €	539	519	131	408	388
Poland	Total costs, 2020 € (Zloty)	612 (2,733)	592 (2,643)	307 (1.369)	305 (1,364)	285 (1,274)
UK	Total costs, 2020 € (pounds)	1.319 (1,175)	1,299 (1,157)	806 (718)	513 (457)	493 (439)
Costs lifetime period^h^ ^,^^k^						
Denmark	Total costs (base case)^i^, 2020 € (DKK)	520,102 (3,884,138)	–	518,366 (3,871,175)	1,736 (12,963)	–
	Total costs (scenario)^j^, 2020 € (DKK)	520,023 (3,883,548)	–	518,366 (3,871,175)	1,657 (12,373)	–
Spain	Total costs (base case)^i^, 2020 €	349,955	349,921	349,631	323	290
	Total costs (scenario)^j^, 2020 €	349,876	349,837	349,631	245	206
Poland	Total costs (base case)^i^, 2020 € (Zloty)	89,627 (399,025)	89,712 (399,401)	89,373 (397,892)	254 (1,133)	339 (1,509)
	Total costs (scenario)^j^, 2020 € (Zloty)	89,463 (398,294)	89,544 (398,653)	89,373 (397,892)	90 (402)	171 (761)
UK	Total costs (base case)^i^, 2020 € (pounds)	336,292 (299,438)	336,194 (299,351)	335,645 (298,862)	647 (576)	549 (489)
	Total costs (scenario)^j^, 2020 € (pounds)	336,417 (299,549)	336,326 (299,468)	335,645 (298,862)	772 (687)	681 (606)
Cost-effectiveness^h^ ^,^^k^						
Denmark	ICUR (base case)^i^ ^,^^l^, 2020 € (DKK)				158,798 (1,185,909)	–
	ICUR (scenario)^j^ ^,^^l^, 2020 € (DKK)				49,626 (370,610)	–
Spain	ICUR (base case)^i^ ^,^^l^, 2020 €				172,789	50,108
	ICUR (scenario)^j^ ^,^^l^, 2020 €				43,562	21,401
Poland	ICUR (base case)^i^ ^,^^l^,2020 € (Zloty)				22,915 (102,018)	Control dominates
	ICUR (scenario)^j^ ^,^^l^,2020 € (Zloty)				1,596 (7,107)	5,373 (23,920)
UK	ICUR (base case)^i^ ^,^^l^, 2020 € (pounds)				14,607 (13,006)	13,726 (12,222)
	ICUR (scenario)^j^ ^,^^l^, 2020 € (pounds)				9,991 (8,896)	9,149 (8,146)

a Different statistical tests were performed. Generalized estimation equations were used to analyze the EQ-5D-5L utilities and linear mixed models were used to quantify the differences in effects between the PP/PN and control of all other health outcomes.

b *p*<0.01 significantly change from baseline.

c *p*<0.05 significant difference between groups.

d *p*<0.05 significantly change from baseline.

e *p*<0.01 significant difference between groups.

f Quality of life score.

g ©Laboratory for the Study of Human Ingestive Behavior, The Pennsylvania State University.

h Discounted results were presented.

i Base case: Point estimates of BMI as observed from the trials were used as input in the model.

j Scenario: The lower level of the 95% confidence intervals from the effect in BMI was used as input in the model.

k All costs were then converted from 2020 national currency to 2020 Euros using the following exchange rates: 1 DKK = 0.134 Euro, 1 Zloty = 0.225 Euro, 1 pound = 1.123 Euro.

l WTP thresholds: Denmark; €47,817 per QALY gained (357,100 DKK), Spain; €30,000 per QALY gained, UK; €22,461 per QALY gained (20,000 pounds), Poland; €38,430 per QALY gained (171,092 Zloty).

BMI, body mass index; CI, confidence interval; cm, centimeter; Cum, cumulative; DKK, Danish krone; DSAT; diet satisfaction questionnaire; EQ-5D, EuroQol five-dimension questionnaire; ICUR, incremental cost-utility ratio; IHD, ischemic heart disease; kg, kilogram; MEDAS, Mediterranean diet score; m, meter; OWLQOL, Obesity and Weight Loss Quality of Life; PN, personalized nutrition intervention; PP, personalized plan intervention; QALY, quality-adjusted life year; SE, standard error; UK, United Kingdom.

Predicting long-term outcomes based on short-term effects on BMI and utilities revealed that generally both PP and PN interventions led to improved lifetime health outcomes compared to the control group, translating into potential benefits such as fewer years with diabetes, increased life expectancy, and lifetime health (QALYs). However, as Poland showed contrasting effectiveness results over the trial period, PN also had worse lifetime health outcomes compared to control (e.g., −0.015 QALYs) in base-case scenario. Scenario analyses, using the lower 95 percent confidence limit of short-term effectiveness on BMI (i.e., −0.45 kg/m^2^), revealed increased QALYs for PN compared to control (+0.032), consistent with findings in other countries. More details on health outcomes can be found in Table 3 and in published materials (26–29;46–48).

### Costs and economic evaluation

The interventions (PP and PN) had higher costs compared to the control over the trial period, with Denmark showing the highest costs (see Table 3). Supplementary Tables S4a–d provide further details on the intervention costs. Over a lifetime horizon, costs were considered from an extended societal perspective, including obesity-related disease costs, unrelated medical costs, nonmedical costs, informal care costs, and productivity costs. In summary, lower costs related to diabetes, IHD, and stroke were offset by higher costs in other areas (i.e., unrelated medical costs, nonmedical costs, and informal care) due to increased life years resulting from the interventions. Depending on the chosen willingness-to-pay threshold and the specific intervention (PP or PN), some interventions were deemed cost-effective, such as PP and PN in the UK and PP in Poland. Scenario analyses revealed additional cost-effective interventions, including PN in Spain and PN in Poland (see Table 3 and published materials (27–29) for more details). Given the high prevalence of overweight and obesity, personalized nutrition interventions would have a substantial budget impact.

### Ethical aspects

This HTA included an examination of ethical issues. The PREVENTOMICS interventions demonstrated a favorable benefit–harm balance, as they showed no significant harms (safety domain) but some improvements in clinical effectiveness (effectiveness domain). Moreover, the interventions respect individual autonomy, human dignity, human rights, and participants’ privacy and integrity. However, health inequality may arise if these interventions are not reimbursed by a third party and may thus be necessary to prevent disparities between wealthier and poorer individuals. More specifically, lower-income individuals generally have poorer diets and higher disease burdens, while higher-income individuals have better access to the interventions (49). Additionally, older individuals may face challenges in using the interventions due to digital illiteracy or lack of suitable mobile phones (see Supplementary material S2 for more details).

### Organizational aspects

In general, the PREVENTOMICS interventions were considered supplementary to the existing work processes of professionals such as nutritionists or dieticians. Professionals were likely to be familiar with the use of apps to document health behaviors but were asked to perform additional tasks related to genetic and metabolic sampling, which they usually do not do. Besides guidance on sampling for genetics and metabolomics, minimal training or education is expected (see Supplementary Table S3). However, besides the comparable study design in the UK and Poland, the (cost)-effectiveness results were not consistent. One possible explanation is that the UK utilized a more didactic approach for providing recommendations, resulting in better outcomes. Providing training to professionals on delivering information may therefore optimize results.

Personalized nutrition requires that participants undergo tests, which might decrease their enthusiasm. However, an app to document food habits and other information could help maintain their motivation. Overall, participants generally accepted the PREVENTOMICS interventions well, despite some difficulties in app usage, particularly in the UK and Poland. However, most problems were solved or had minimal impact. More details and examples can be found in Supplementary material S3.

### Patients and social aspects

Understanding the experiences of overweight or obese individuals is crucial for the success of PREVENTOMICS interventions. Farrell et al. (50) found that people with obesity experience negative issues, such as emotions, traumas, restrictions in movements, stigma, and lack of respect. The DSat-28 results indicated slight increases in diet satisfaction for almost all intervention groups compared to baseline (see Table 3). Additionally, a DCE study revealed willingness to choose personalized nutrition interventions, with total expenditure being the most important factor influencing peoples’ preferences (34). Behavioral reminders were not highly valued. The DCE study also showed participation rates for specific scenarios, including scenarios somehow similar to PREVENTOMICS interventions, and revealed rates varying from 26 percent to 49 percent across countries and interventions (34). Moreover, a UK cohort study revealed substantial variations in genetic testing preferences, which tests are also needed in personalized nutrition interventions, between white and ethnic minority individuals, with the white cohort being twice as likely to undergo genetic testing (51).

Gaining user trust is crucial for intervention success, emphasizing the importance of transparent and simple explanations of interventions and their benefits (D1.2 (“Consumers Report”)). In the Danish trial, 50 percent of the participants were excited to be part of the study and inspired to eat more vegetarian-based food, but they also missed familiar meals and felt isolated (D5.3 (“Report on the outcome of each intervention study”). In the Spanish trial, participants criticized time-consuming shopping lists. In the UK and Poland, participants felt cared for by healthcare professionals, and some participants felt better during the dietary intervention than before. However, some mentioned that adhering to the diet was more time-consuming and expensive than their previous diet.

### Legal aspects

Personalized nutrition lacks specific legal regulations due to its multifaceted nature (which includes aspects such as advice, testing and foods), making legislation fragmented (52;53). In other words, personalized nutrition interventions can be categorized as “health” or “lifestyle” intervention or “food” or “medicine,” affecting the applicable rules and regulations (53). Röttger-Wirtz and De Boer (53) analyzed food laws and showed for example that, it is often unclear whether certain nutrigenomic or nutrigenetic effects should be classified as health optimizing, health maintaining, or disease preventive effects. Classifying it as disease preventive, results, for example, in regulating the intervention as a medicinal product, rather than governed by food laws.

There are legal requirements that apply to all personalized nutrition interventions, including the General Data Protection Regulation (GDPR) for personal data. GDPR guidelines were prioritized in the PREVENTOMICS interventions by ensuring anonymization. Moreover, CE marking is required under the current medical device regulation for the European market, as interventions like PREVENTOMICS are classified as in vitro diagnostic medical devices (54). For more details, see Supplementary material S4.

## Discussion

This study aimed to assess the PREVENTOMICS interventions in a pre-market phase with the HTA Core Model to inform development and implementation decisions. Conducting an “early HTA” is an effective method to identify and address potential issues regarding market access and reimbursement (55). The different domains showed that approaches like PREVENTOMICS to reduce overweight and obesity are needed. Moreover, people express willingness to use these interventions (34), though certain groups (i.e., white individuals) exhibit a higher likelihood of genetic testing than others (i.e., ethnic minority individuals) (51). Furthermore, our findings indicate that PREVENTOMICS interventions entail low safety risks and require minimal training. While their implementation may require some challenges at the organizational level, the trials showed that they are resolvable.

PREVENTOMICS interventions could have favorable effectiveness results; small short-term effects observed during the trials could translate into long-term health benefits (1;2). Results align with other studies; see Aldubayan et al. (26) for comparison of PREVENTOMICS effectiveness results with other studies. Additionally, Galekop et al. (56) found that personalized nutrition interventions often led to incremental QALYs between 0 and 0.1, comparable with our study findings. While the effects observed are small, most effects are clinically meaningful (requiring a minimum 0.03 difference in utility score) (57;58). However, in Spain, short-term effects resulted in minimal long-term benefits for both PP and PN interventions compared to control (incremental QALYs of 0.002 and 0.006, respectively), contrasting with other countries where incremental QALYs were at least 0.01. Between country differences may stem from the diverse interventions and populations, including cultural differences and targeted weight classifications. For example, Aune et al. (59) demonstrated a J-shaped relationship between BMI and all-cause mortality, potentially explaining the lower effect observed in Spain, which encompasses the general population, including those with normal weight, unlike other countries where studies focused on people with overweight and obesity.

Although clinical trials on technology-based and personalized nutrition interventions often feature small sample sizes and short follow-ups (7;60), leading to effectiveness and parameter uncertainties in cost-effectiveness analyses, Hogervorst et al. (61) suggested improving data quality and quantity to reduce uncertainty, which for PREVENTOMICS interventions could be achieved by longer and larger trials. Our cost-effectiveness analyses explored the potential health benefits of the interventions in the scenario analyses and revealed promising cost-effectiveness results for the interventions in Spain, the UK, and Poland.

The use of PREVENTOMICS interventions would likely increase both short-term and lifetime costs, which raises various questions. First, our findings support the literature indicating that personalized nutrition is more often used by motivated and wealthier individuals (53), particularly when out-of-pocket payments are required. This raises ethical concerns, as personalized nutrition can exacerbate health inequality, given that individuals with lower socioeconomic status often have poorer diets and higher disease burdens but may struggle to afford these interventions (49). Therefore, third-party reimbursement for effective personalized nutrition interventions is crucial. However, budget constraints may prevent decision-makers to reimburse interventions for the whole target population. It may therefore be advisable to consider reimbursing effective personalized nutrition interventions only for subpopulations with the highest health or economic burden (e.g., severely obese) (62). Alternatively, partial subsidies could be provided, covering specific components of the interventions, such as testing or mobile app costs.

Additionally, we recommend that stakeholders, such as policymakers, should collaborate to develop a cohesive legal framework that fosters consumer trust, engagement and enables personalized nutrition to reach its full potential (53;63). Furthermore, policymakers, together with developers, should focus on addressing the concerns of ethnic minority individuals, specifically regarding employment repercussions of genetic tests (51), ensuring inclusivity and avoiding exclusion due to information shortages. Moreover, despite the ending of the EUNetHTA Joint Actions by September 2023, collaboration on HTAs is recommended between countries to keep track of the fast-changing field of personalized nutrition and to produce timely HTA information for decision-makers. The new “regulation on HTA” is expected to support this future collaboration (64).

This HTA has several limitations. First, as the HTA Core Model was not designed for personalized nutrition interventions (25), additional domains or assessment elements may be needed. Becla et al. (65) highlighted the importance of ethical, organizational, social, and legal aspects in personalized health care and suggested rethinking the “gold standard” of large trials and instead considering “personal evidence.” Moreover, Von Huben et al. (66) identified inconsistencies in current HTA frameworks for digital health tools, suggesting the inclusion of digital-specific content in existing or new elements of the HTA Core Model. More specifically, potential additions to the HTA assessment of PREVENTOMICS interventions could be the consideration of device features like size, battery life, operating system, technical support, and connectivity (assessment element ID B0007 should be modified). Moreover, adding new assessment elements could be considered, for example, DHT08 in the safety domain (66): “how well are updates/continuity of digital health technologies managed?” While we believe all essential aspects are covered in our HTA, future research should analyze more aspects for a more comprehensive overview of digital tools in personalized nutrition interventions.

Second, we obtained expert opinions in this HTA without a systematic approach and we did not fully follow the recommended EUnetHTA methodological framework. Nonetheless, we believe that our approach identified the most critical issues in personalized nutrition interventions.

Third, this HTA primarily focused on BMI as (short-term) outcome measures, but other health outcomes such as waist circumference, blood glucose, systolic blood pressure, or LDL cholesterol might even be more important (67;68). However, there is limited literature on translating short-term changes in these outcomes into lifetime estimates of disease risk, health outcomes, and costs (32).

In addition to previously mentioned future research suggestions, another recommendation is to extend this HTA by using multiple criteria decision analysis (MCDA) to systematically evaluate and rank ideas based on weighted criteria (69). Since MCDA can identify the relative importance of different criteria, this method can help to maximize societal value when resources are allocated (69).

## Conclusion

In conclusion, our HTA emphasizes the relevance of evaluating personalized nutrition interventions beyond costs, effects, and economic aspects by addressing different (related) issues. While PREVENTOMICS interventions exhibit potential (cost)-effectiveness, developers should prioritize gathering additional evidence through longer and larger-scale trials. Addressing organizational issues and early discussions with third-party payers about reimbursement options are recommended for developers. Additionally, policymakers, together with developers, should work on collecting and providing accessible and comprehensive information (e.g., on genetic testing) for all ethnic groups. Moreover, a cohesive legal framework and a system-wide collaboration among stakeholders, including European HTA, are needed, prior to making implementation decisions.

## Supporting information

Galekop et al. supplementary material Galekop et al. supplementary material

## Supplementary material

The supplementary material for this article can be found at http://doi.org/10.1017/S0266462324000060.
