# Supplementary material for: A health technology assessment of personalized nutrition interventions using the EUnetHTA HTA Core Model
Source: Int J Technol Assess Health Care. 2024 Mar 6;40(1):e15. doi: 10.1017/S0266462324000060 (PMC11569966; doi:10.1017/S0266462324000060)
Supplement: Galekop et al. supplementary material [file S0266462324000060sup001.pdf]

Supplementary files

Contents

Supplementary Table 1: deliverables ... 2
Supplementary Table 2: obesity classification ... 3
Supplementary Figure 1: User Journey ... 3
Supplementary Table 3: trainings and tools ... 4
Supplementary Figure 2a-2d: study designs ... 5
Supplementary Text 1: reimbursement ... 7
Medical nutrition ... 7
Medical nutrition therapy (MNT) ... 7
Prevention in general, including lifestyle interventions ... 8
Digital health ... 8
Supplementary Table 4a-4d: intervention costs ... 10
Supplementary Text 2: ethical issues ... 12
Benefit-harm balance ... 12
Autonomy ... 12
Respect for persons ... 12
Justice and equity ... 12
Legislation ... 13
Ethical consequences of the HTA ... 13
Supplementary Text 3: organizational issues ... 14
Health delivery process ... 14
Structure of health care system ... 15
Process-related costs ... 15
Management ... 15
Culture ... 16
Supplementary Text 4: legal aspects ... 17
Privacy of the patient ... 17
Equality in health care ... 17
Ethical challenges of existing legislations ... 18
Authorization and safety ... 18
Ownership and liability ... 18
Regulation of the market ... 18
References ... 20

Supplementary Table 1: deliverables

Table 1: (Reference) information of the deliverables used in the health technology assessment

Deliverable name	Part of (1) work package (WP) and (2) task (T)	Actual date of delivery	Used in domain
Deliverable 1.2 (‘Consumers Report’) (published)*	(1) WP1: Business modelling<br>(2) T1.2 Consumers and Business Requirements Alignment	31-07-2019	Health problem and current use of technology (CUR)<br>Ethical analysis (ETH)<br>Patients and Social aspects (SOC)
Deliverable 4.1 (‘PREVENTOMICS platform design’)	(1) WP4: PREVENTOMICS personalized nutrition integration<br>(2) T4.1 PREVENTOMICS platform design and communication interfaces specification	31-10-2019	Description and technical characteristics of technology (TEC)
Deliverable 5.3 (‘Report on the outcome of each intervention study’)	(1) WP5: Consumer-centered interventions<br>(2) T5.4: Deployment of the intervention	21-07-2022	Description and technical characteristics of technology (TEC)<br>Clinical effectiveness (EFF)<br>Costs and economic evaluation (ECO)<br>Patients and Social aspects (SOC)
Deliverable 5.4: (‘Overall performance of PREVENTOMICS service’)	(1) WP5: Consumer-centered interventions<br>(2) T5.5 Validation of the PREVENTOMICS approach and business cases	23-06-2022	Clinical effectiveness (EFF)
Deliverable 6.1 (‘Ethical framework’)	(1) WP6: Regulatory, Economic and Health impact<br>(2) T6.1 Regulatory, Ethics and Gender aspects	29-04-2019	Legal aspects (LEG)
Deliverable 6.2 (‘Regulatory framework’)	(1) WP6: Regulatory, Economic and Health impact<br>(2) T6.1 Regulatory, Ethics and Gender aspects	28-04-2020	Legal aspects (LEG)
Deliverable 6.4 (‘Cost-effectiveness analyses results’)	(1) WP6: Regulatory, Economic and Health impact<br>(2) T6.3 Cost-effectiveness	25-07-2022	Clinical effectiveness (EFF)<br>Costs and economic evaluation (ECO)<br>Patients and Social aspects (SOC)
Deliverable 7.2 (‘Data management plan’)	(1) WP7: Market impact management and dissemination<br>(2) T7.4 Knowledge and Data Management	30-04-2019	Ethical analysis (ETH)<br>Legal aspects (LEG)
Deliverable 7.4 (‘PUDR’)	(1) WP7: Market impact management and dissemination<br>(2) T7.1 IPR Management and Exploitation	31-05-2020	Legal aspects (LEG)
Deliverable 7.5 (‘Final plan for the Use and Dissemination of Results-PUDR’)	(1) WP7: Market impact management and dissemination<br>(2) T7.1 IPR Management and Exploitation	31-10-2021	Health problem and current use of technology (CUR)<br>Description and technical characteristics of technology (TEC)<br>Legal aspects (LEG)
Deliverable 9.1 (‘Requirement №1 – Humans - Interventional studies‘)	(1) WP9 Ethics requirements	26-07-2022	Description and technical characteristics of technology (TEC)

***Published online:** https://preventomics.eu/deliverables/#1593502709004-84c73ce5-2fe4

Supplementary Table 2: obesity classification

Table 2: Obesity classification (1)

BMI (kg/m^2)	Classification
< 18.5	Underweight
18.5 to <25	Healthy weight
25.0 to <30	Overweight
30.0 or higher	Obesity
30 to <35	Class 1
35 to <40	Class 2
40 or higher	Class 3 (severe obesity)

Supplementary Figure 1: User Journey

Figure 1: PREVENTOMICS User Journey (2)

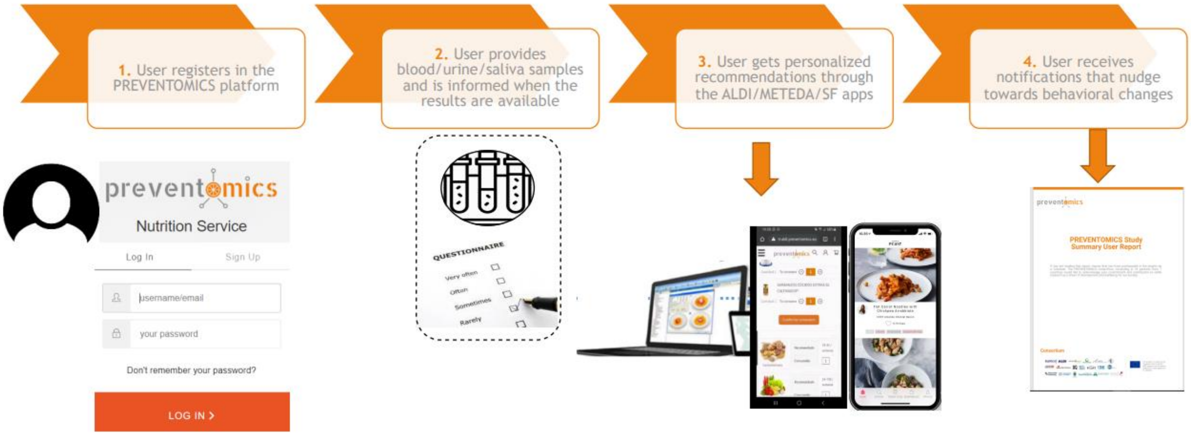

Supplementary Table 3: trainings and tools

Table 3: Summary of main findings on training and tools needed for PREVENTOMICs interventions

	Country	Training	Tools
**Participants (volunteers/patients receiving intervention)**	*Denmark*	No training required. However, it is good to highlight the importance of good food and to give them background information of personalized nutrition.*	Mobile phone, meals, internet connection
	Spain	No training required. However, it is good to highlight the importance of good food and to give them background information of personalized nutrition.*	Mobile phone, internet connection
	Poland/UK	When dietary recommendations are given by the dietician/nutritionist through the MetaDieta app, participants can use this app as well. Training for this app can be given by the dietician/nutritionist, to make sure the participant understands it correctly.*	Mobile phone, internet connection
**Professionals**	*Denmark*	Training for SimpelFeast (or other companies that will use the intervention when it might be on the market). This might be about the background information of personalized nutrition, but also more technical stuff (e.g., how to integrate the PREVENTOMICS platform with the SimpleFeast app).**	All necessities for doing blood, urine, and saliva tests (needles, samples etc.). And all necessities for measuring all anthropometric measures (scale, measuring tape etc.).
	Spain	Training for Aldi professionals (or other supermarkets when they are interested in this intervention). This might be about the background information of personalized nutrition, but also more technical stuff (e.g., how to integrate the PREVENTOMICS platform with the ALDI app).**	
	Poland/UK	Training for nutritionists/dietician about the use and importance of the application (e.g., lectures on genetic changes in obesity (see Deliverable 7.5 ('Final plan for the Use and Dissemination of Results-PUDR')).**	All necessities for doing blood, urine, and saliva tests (needles, samples etc.). And all necessities for measuring all anthropometric measures (scale, measuring tape etc.). Moreover, dietician consultation rooms and all that is needed for this consultation (e.g., gloves).
**Service exploitator (someone who can store and analyze the data).**		Training on how to use and maintain the PREVENTOMICS platform. Moreover, how to integrate this with different business cases.	Computers, internet connection. Materials to analyze the data.

**Training might be needed if it is asked from the participants to take blood, urine, and salvia samples themselves.*

*** Training might be needed if professionals are asked to take the blood, urine, and salvia samples from the participants and if they are not used to do this.*

Supplementary Figure 2a-2d: study designs

Figure 2a: Study design Denmark (Deliverable 9.1 ('Human Interventional Studies'))

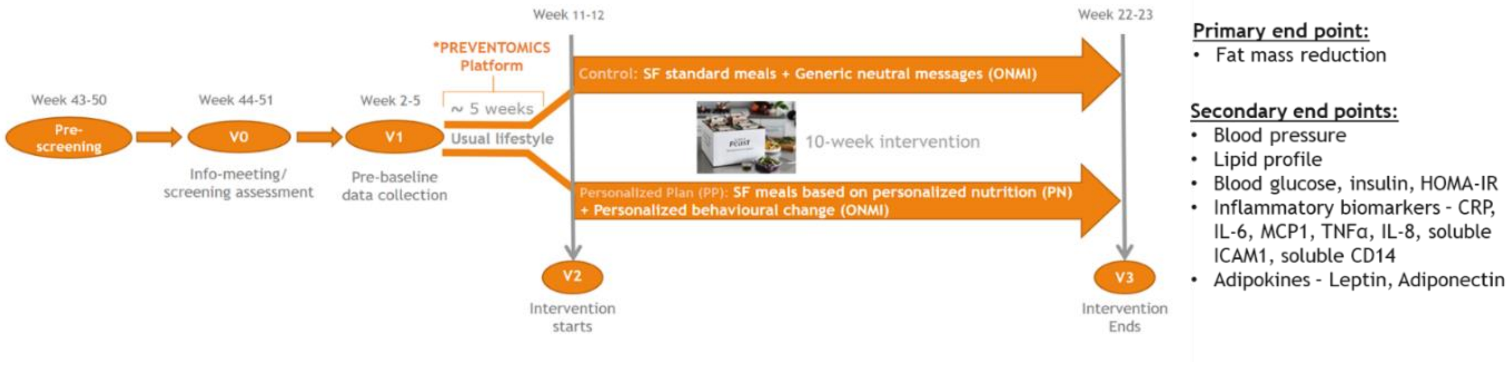

Figure 2b: Study design Spain (Deliverable 9.1 ('Human Interventional Studies'))

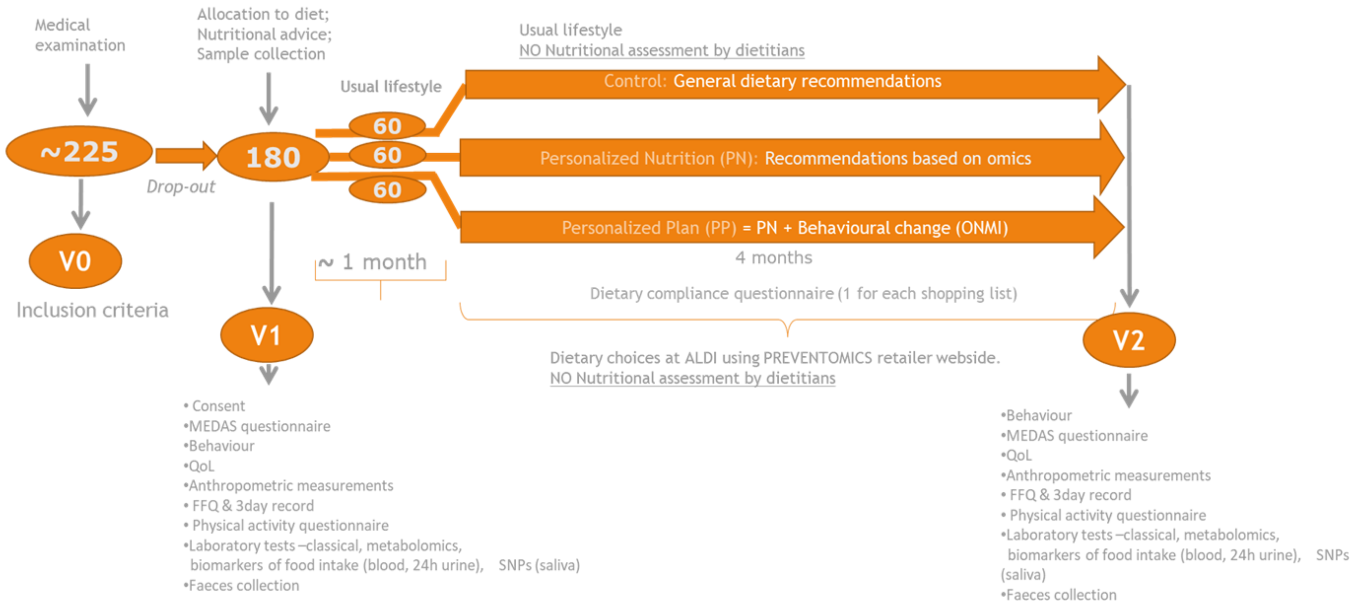

Figure 2c: Study design Poland (Deliverable 9.1 ('Human Interventional Studies'))

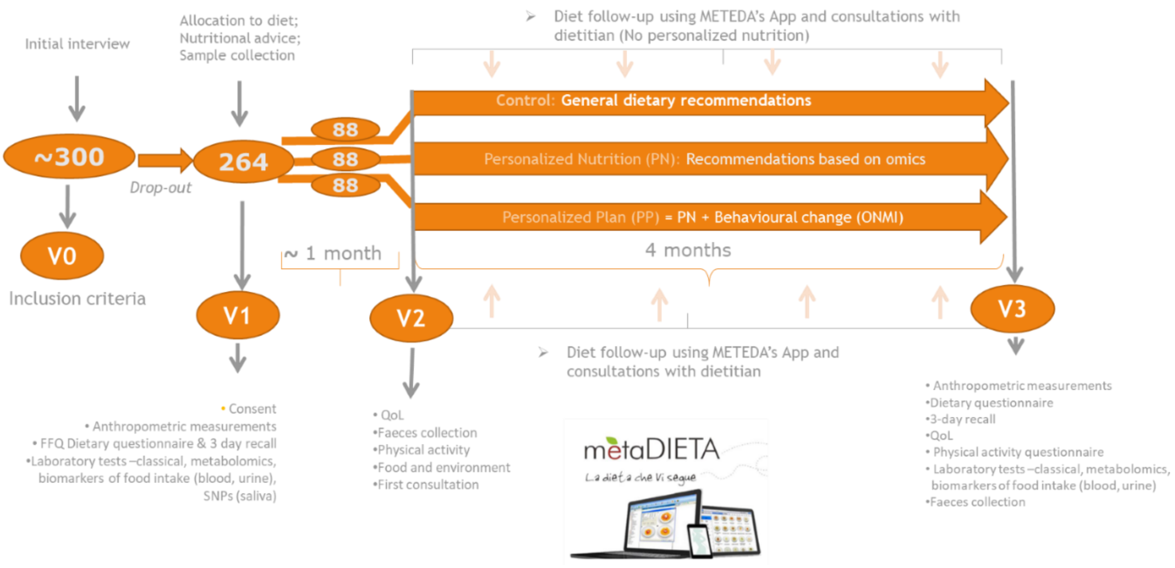

Figure 2d: Study design UK (Deliverable 9.1 ('Human Interventional Studies'))

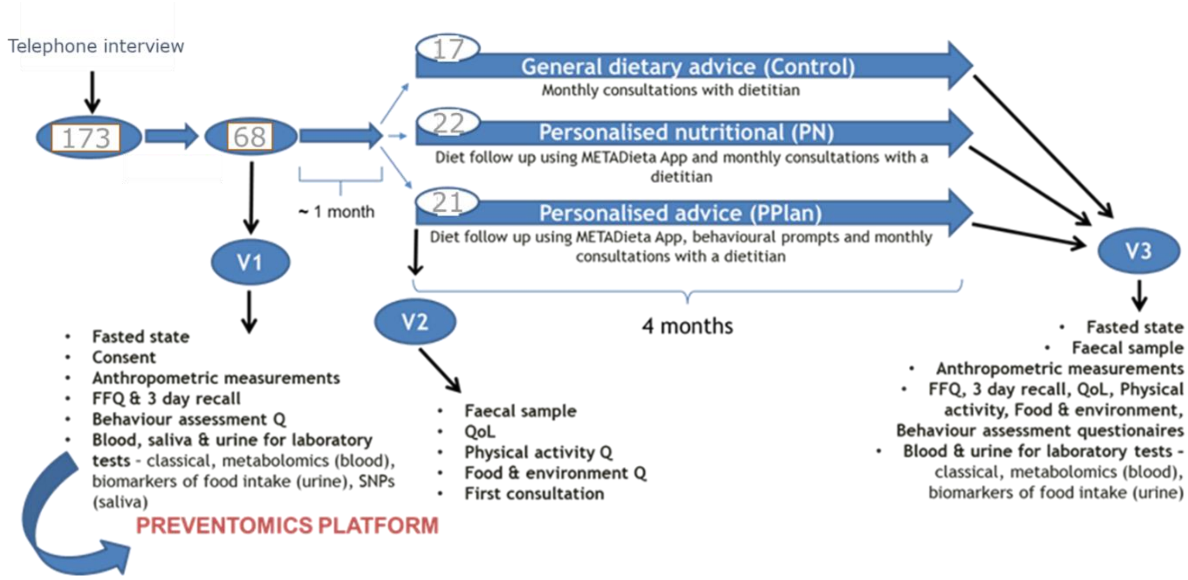

Supplementary Text 1: reimbursement

Text 1: An overview of literature and other online sources on reimbursement regulations of personalized nutrition and related areas in Europe and the United States

Since results of Health technology assessments (HTA) may eventually be used in making treatment guidelines or to inform policy makers in their reimbursement decisions (i.e., include or exclude a treatment in a benefits package) (3), it is important to say something about reimbursement in this HTA as well. Although there is increasing literature and information available on the HTA and reimbursement procedures about pharmaceuticals, there is often less known about these procedures for non-pharmaceuticals (4,5). Moreover, it is not only the procedure that is often unknown, non-pharmaceuticals, such as nutrition related products, are often not reimbursed at all. While there are examples of nutrition interventions that were reimbursed, food products and nutrition interventions are typically not reimbursed by a third-party payer (3,6). Instead, users need to pay out-of-pocket. In this Supplementary Text, we highlight several findings in the literature about the reimbursement of this type of interventions. Moreover, examples of related areas, such as medical nutrition, medical nutrition therapy (MNT) for diabetes type 2, lifestyle interventions, and digital health are given. It must be noted that the information provided below are specific examples in countries, but that each country has its national and local policies.

Medical nutrition

Medical nutrition is defined as food for special medical purposes/medical food by Perugini et al.(5), who studied the coverage and reimbursement of medical nutrition in different countries. It is not used for prevention purposes, but instead used to treat nutrition-related disorders and conditions such as malnutrition. It comprises two specific types: enteral nutrition and parenteral nutrition. Enteral nutrition includes oral nutritional supplements and enteral tube feedings into the digestive tract and its use is regulated via food for special medical purposes/medical food. Parental nutrition is administered intravenously and is regulated by pharmaceutical legislation. It was found by Perugini et al.(5) that most countries have limited reimbursement/coverage for medical nutrition, especially in the outpatient/community setting. Moreover, these policies were often outdated or there was a lack of HTA on medical nutrition at all (i.e., France and Brazil were the only countries with formal HTA procedures) (5).

In Denmark, patients who receive nutritional therapy upon discharge are subject to the rules for dispensing pharmaceuticals (7). In the case of parenteral nutrition, reimbursement regulations are based on the clinical diagnosis rather than the patient's nutritional status. There is a lack of clarity regarding financial responsibility, including reimbursement for parenteral nutrition after discharge. Palliative care patients and those with short bowel syndrome have a designated reimbursement system, but it is unclear for other diagnoses (7).

Medical nutrition therapy (MNT)

A common approach in the prevention of type 2 diabetes is MNT (8), which is defined as “nutritional diagnostic, therapy, and counselling services for the purpose of disease management which are furnished by a registered dietician or nutrition professional…”(8). In the US, a physician referral is needed to get MNT reimbursed by a payer like Medicare (national health insurance program). However, current procedural terminology and billing procedures for MNT vary and are interpreted differently by carriers and billing agencies, within government-funded programs and private sector insurance plans. Moreover, the US Preventative Services Task Force recommends screening for abnormal blood glucose to be part of cardiovascular risk assessments (8). This task force focuses on adults aged 40-70 years who are overweight or obese. If they are diagnosed with abnormal blood glucose, the clinician should offer them intensive behavioral counselling interventions to promote a healthy diet plan and increase physical activity. However, coverage is not guaranteed by all plans. Additionally, nutrition services, including diabetes education by registered dietician nutritionists, are also often part of a bundled payment system in acute care settings. There is a growing adoption of alternative payment models in the US, which creates the opportunity to support nutrition services to prevent diabetes based on factors such as their cost-effectiveness (8).

In Poland, generally healthy people, or obese people without significant complications seek dietary advice, personalized or not, only in private facilities and at their own expense (9,10). Since October 2022, some

patients can receive free dietary advice through the National Heath Fund. Eligible patients include diabetic patients, patients treated in cardiology, patients seen in pulmonary/allergy clinics, and patients with thyroid diseases.

Prevention in general, including lifestyle interventions

Overall, general practitioners in Europe experience a barrier to use health promotion in clinical practice (11). One of these main barriers is the lack of reimbursement in these activities. Participants in another study of obesity management in Europe (12) mentioned the need for reimbursement of dieticians, physical activity professionals as well as psychologists and the need for better promotion of healthy lifestyles. Insurance companies need to be involved if there is no national health service that can provide what is needed.

A cross-sectional survey study of lifestyle medicine (LM) practitioners in the US (13) reported results that were similar to those found in the two above mentioned studies in Europe (11,12). LM is defined here as “a clinical discipline in which practitioners and the entire healthcare team treat many common non-communicable chronic diseases using health behavior change as the foundation of care”. This could include interventions such as changing the eating pattern, regular physical activity, stress management and more. This study reported that 55% of practitioners were unable to receive reimbursement for their LM practice. Among the 471 survey respondents who answered the question about how to make LM practice easier, several suggestions were offered. Among others, these included: overall reimbursement, reimbursement for more time spent with patients and reimbursement for the extended care team (13).

Zwaagstra Salvado et al.(14) studied the links between reimbursement and prevention in the Netherlands. They found that there is not just one reimbursement scheme available that will stimulate all levels of prevention, but that different types of reimbursement work well for different preventive services. For example, prevention activities that are easy to specify could benefit from a volume incentive (as an example of fee for service). Interventions that are not easily specified, such as providing education on lifestyle factors, could better work with population-based capitation reimbursement.

One specific example of an intervention that is reimbursed for Dutch citizens with overweight or obesity per January 2019 is the combined lifestyle intervention (CLI) (15). These are multicomponent interventions lasting two years, consisting of interactive sessions with care professionals. Moreover, it is tailored to the participant’s needs. More information can be found in the literature (15).

Digital health

The market of digital health solutions is a rapidly growing sector, but reimbursement from public payers is often lacking (16). More specifically, in the UK and the Netherlands, there is no national-level reimbursement framework for low-risk health apps (17). Individual trusts/CCGs can cover these kinds of apps in the UK and in the Netherlands individual insurance companies can cover apps or can jointly purchase them. Digital health solutions are currently not evaluated within an HTA framework in the Netherlands. However, in the UK, NICE has developed a digital health technology framework to assess digital health solutions (17). The UK also has an NHS Apps library, collecting all health apps that have been assessed against national standards and have been proven to be safe and secure. However, the addition of an app to this catalogue thus does not mean that funding or reimbursement will necessarily follow. It is recommended that this link with funding, reimbursement and/or coverage increases (17).

Compared to other European counties, the pathway of reimbursement of digital solutions is quite mature in the UK (16). For example, in Spain there are numerous highly independent regional payers, each with their own unique reimbursement pathways and evidence requirements for digital solutions. This market is therefore a challenge to tackle (16).

In Germany, the parliament introduced the digital healthcare act (Digitales Versorgungsgesets, DVG) by the end of 2019 (18). This act describes a pathway for the reimbursement of digital health apps (i.e., digitale Gesundheitsanwendung, DiGA). In other words, 90% of the German population in insured by the statutory health insurance (SHI) and the DVG grants individuals with SHI the right to receive benefits for certain DiGA.

This means that insurers will cover the expenses associated with utilizing these DiGA (19). However, coverage benefits will only be granted if the DiGA meets the following criteria (18,19):

1. Show a beneficial impact on healthcare, either through medical benefits or improvements in healthcare procedures and structures.
2. Categorized as a low-risk medical device (Class I or IIa) in accordance with medical device regulation (MDR).
3. Primarily operates based on digital technology.
4. Serves a medical purpose, such as monitoring, detecting, alleviating, or treating illnesses, or compensating, detecting, relieving, or treating injuries or disabilities for injured individuals or in healthcare provided by service providers.
5. Primarily centered around the patient.

In June 2023, there were already 53 DiGA applications approved for reimbursement (20). DiGAs that were approved focused on psychology, but other therapeutic areas included for example stroke, obesity, and diabetes (20).

Given these various health system structures, funding models, and regulations within and between countries, coupled with heightened scrutiny from payers, providers, and physicians, it is crucial for developers to invest significant time and effort in proving the effectiveness and efficiency of their new treatments if they hope to receive reimbursement on a large scale (16). It is recommended for future research to make a clear overview of reimbursement policies in different countries in Europe about all different areas related to personalized nutrition, by means of an extended literature search and/or online documents of the countries of interest.

Supplementary Table 4a-4d: intervention costs

Table 4a: Danish trial: average intervention costs per participant (2020 €, (DKK))

Components	PP	Control	Difference
**Meals (breakfast & dinner, eaten 6 days per week)**			
Direct costs			
Food costs	2,746 (20,507)	2,746 (20,507)	0
Packaging costs	1,239 (9,253)	1,239 (9,253)	0
Production costs	1,273 (9,507)	318 (2,375)	955 (7,132)
Delivery costs	189 (1,411)	189 (1,411)	0
Indirect costs (25% of direct costs)	1,362 (10,171)	1,123 (8,387)	239 (1,784)
Functional ingredients	5.00 (37.39)	0	5.00 (37.39)
Total meal costs	*6,814 (50,887)*	*5,616 (41,940)*	*1,198 (8,947)*
**Behavioral messages via app**	15 (112)	15 (112)	0
**Access SF app recipes**	21 (155)	21 (155)	0
**PREVENTOMICS platform (storage data + questionnaires maintenance)**	0.81 (6.02)	0.81 (6.02)	0
**Tests (blood, urine, saliva)**			
Omics	383 (2,857)	0	383 (2,857)
Genetics	54 (403)	0	54 (403)
Other (e.g., overhead)	115 (857)	0	115 (857)
Total tests costs	*550 (4,111)*	*0*	*550 (4,111)*
**TOTAL COSTS**	**7,402 (55,277)**	**5,653 (42,215)**	**1,749 (13,062)**

DKK, Danish Krone; PP, Personalized Plan; SF, Simple Feast.

Table 4b: Spanish trial: average intervention costs per participant (2020 €)

Components	PP	PN	Control	Difference PP-Control	Difference PN-Control
**Behavioral messages via app per participant**	10	0	0	10	0
**Behavioral message integration with ALDI microsite + maintenance**	10	0	0	10	0
**Extra costs grocery shopping (eating healthier)**	130	130	130	0	0
**PREVENTOMICS platform (storage data + questionnaires maintenance)**	1.40	1.40	1.40	0	0
**Tests (blood, urine, saliva)**					
Omics	257	257	0	257	257
Genetics	54	54	0	54	54
Other (e.g., overhead)	77	77	0	77	77
Total tests costs	*388*	*388*	*0*	*388*	*388*
**TOTAL COSTS**	**539**	**519**	**131**	**408**	**388**

PN, Personalized nutrition; PP, Personalized Plan

Table 4c: Polish trial: average intervention costs per participant (2020 €, (Zloty))

Components	PP	PN	Control	Difference PP-Control	Difference PN-Control
**Access for participant and center (professional) + maintenance MetaDieta app/software**	30 (134)	30 (134)	0	30 (134)	30 (134)
**Dietician/Nutritionist appointments**	112 (500)	112 (500)	112 (500)	0	0
**Behavioral messages via app per participant**	10 (45)	0	0	10 (45)	0
**Behavioral message via app integration with MetaDieta + maintenance**	10 (45)	0	0	10 (45)	0
**PREVENTOMICS platform (storage data + questionnaires maintenance)**	1.40 (6.23)	1.40 (6.23)	0	1.40 (6.23)	1.40 (6.23)
**Extra costs grocery shopping (eating healthier)**	195 (869)	195 (869)	195 (869)	0	0
**Tests (blood, urine, saliva)**					
Omics	154 (687)	154 (687)	0	154 (687)	154 (687)
Genetics	54 (241)	54 (241)	0	54 (241)	54 (241)
Other (e.g., overhead)	46 (206)	46 (206)	0	46 (206)	46 (206)
Total test costs	*255 (1,134)*	*255 (1,134)*	*0*	*255 (1,134)*	*255 (1,134)*
**TOTAL COSTS**	**612 (2,733)**	**592 (2,643)**	**307 (1.369)**	**305 (1,364)**	**285 (1,274)**

PN, Personalized nutrition; PP, Personalized Plan

Table 4d: UK trial: average intervention costs per participant (2020 €, (pounds))

Components	PP	PN	Control	Difference PP-Control	Difference PN-Control
**Access for participant and center (professional) + maintenance MetaDieta app/software**	30 (27)	30 (27)	0	30 (27)	30 (27)
**Dietician/Nutritionist appointments**	430 (383)	430 (383)	430 (383)	0	0
**Behavioral messages via app per participant**	10 (8.9)	0	0	10 (8.9)	0
**Behavioral message via app integration with MetaDieta + maintenance**	10 (8.9)	0	0	10 (8.9)	0
**PREVENTOMICS platform (storage data + questionnaires maintenance)**	1.40 (1.25)	1.40 (1.25)	0	1.40 (1.25)	1.40 (1.25)
**Extra costs grocery shopping (eating healthier)**	376 (335)	376 (335)	376 (335)	0	0
**Tests (blood, urine, saliva)**					
**Omics**	314 (279)	314 (279)	0	314 (279)	314 (279)
**Genetics**	54 (48)	54 (48)	0	54 (48)	54 (48)
**Other (e.g., overhead)**	94 (84)	94 (84)	0	94 (84)	94 (84)
Total test costs	*462 (412)*	*462 (412)*	*0*	*462 (412)*	*462 (412)*
**TOTAL COSTS**	**1.319 (1,175)**	**1,299 (1,157)**	**806 (718)**	**513 (457)**	**493 (439)**

PN, Personalized nutrition; PP, Personalized Plan

Supplementary Text 2: ethical issues

Text 2: Details on the ethical issues considered in this health technology assessment

This HTA addresses various ethical issues, categorized according to the HTA core model, and supported by existing literature (21–23). These issues were addressed to maximize benefits and minimize potential harms.

In general, the PREVENTOMICS intervention protocols (24–27) were submitted to the Ethics Committees of the centers involved in each of the studies (D7.2 ('Data management plan')). The Ethical Committees assessed the characteristics of the interventions, informed consent for each protocol, the logistics for data management within each site as well as data management procedures that were needed for joint analysis of the information among sites. The ethical standards and guidelines of Horizon 2020 (in particular: EU Directive 95/46/EC; 2002/58/EC and 2006/24/EC) have been rigorously applied, regardless of the country in which the interventions were carried out. Furthermore, an Ethics Board comprised of representative persons from the partners involved in volunteers' recruitment and sensitive data handling, oversaw evaluating the compliance with the applicable regulations in terms of protection of rights and safety of subjects that contributed with the data used in the project.

Benefit-harm balance

When looking at the "benefit-harm balance", there were measures showing that personalized nutrition could be effective (see Table 3 manuscript), although the effects are not very large, with no major harms (see safety domain). Moreover, is possible that personalized nutrition could result in participants making healthier choices for other people in their lives. For example, a person who cooks for "others" (e.g., relatives) may choose to cook (healthier) foods for them as well. This might influence the eating pattern of the "others" as well. However, this would not be seen as a direct result of personalized nutrition itself. Additionally, the technology and evidence generation for assessing personalized nutrition are unlikely to have hidden or unintended consequences.

Autonomy

The intervention generally had no impact on individual autonomy, supported by different reasons. First, the PREVENTOMICS interventions was not offered to individuals that were vulnerable (and is also not aimed to be offered to vulnerable individuals when on the market), so people are always able to give informed consent. Second, all participants received an information folder before the informed consent was given and always had the right to withdraw at any time. Third, individuals that took part in these interventions were required to be more pro-active about food habits, particularly when the individual receives food recommendations. Last, since it is very common for participants to become less compliant over time, withdrawal is highly unlikely to adversely affect the doctor-patient relationship, or in the case of the pilot in the UK and Poland: the dietician-participant relationship.

Respect for persons

The use of a personalized nutrition intervention is very unlikely to have any adverse effect on human dignity or on participant integrity. One issue that will be respected is the participant's dietary preferences, which may or may not be based on religious or moral beliefs. Therefore, for example, vegetarians will never be told to eat meat. People that prefer to eat meat, can still eat meat in the Danish pilot during lunch or on their seventh day when they need to take care of their own meals.

Justice and equity

The implementation of personalized nutrition is unlikely to significantly affect the distribution of healthcare resources. The costs associated with it are generally not excessively high, although this may vary. Furthermore, personalized nutrition interventions do not require significant reallocation of resources, as they typically involve minimal training and infrastructure requirements. However, there are factors that could prevent a group or individual from gaining access to the PREVENTOMICS interventions. One factor would be digital literacy (or really illiteracy), since the PREVENTOMICS interventions require digital skills and the use of a smartphone (e.g., for older individuals). Another related issue that could have a negative effect on the access to the interventions is the educational status. See for example D1.2 ('Consumers Report'), in which it is found

that the “level of education” best explains the use of (or registration in) a health platform. People with a high level of education are more likely to use or register in these platforms (44%) than respondents with an average level of education (35%) and basic level of education (14%). In other words, PREVENTOMICS aims to be useful for everyone as a preventive tool (health and less healthy), but inequality might arise when interventions will not be reimbursed by a third party. Although people with a lower socioeconomically disadvantage appear to have poorer diets and higher disease burdens, the interventions might be more accessible to people with a greater socioeconomic advantage (28).

Legislation

Personalized nutrition is unlikely to have any impact on the realization of basic human rights or lead to any new ethical challenges at this time. Maybe In the distant future, sophisticated versions of personalized nutrition could result in dramatic health improvements, which may lead to new and unique ethical challenges (e.g., genetic testing).

Ethical consequences of the HTA

There are no obvious ethical consequences of the choices made in the pilot studies. That is, all studies used fairly standard and widely accepted endpoints and cut-off values. Moreover, no obvious ethical problems related to the data or assumptions in the economic evaluations were made. One important reason to conduct the assessment now (i.e., early HTA) is to explore the potential value of personalized nutrition based on the results of the different pilot studies. In that regard, the aim of the assessment was to support developers of personalized nutrition and not perse to support a stop-go decision for implementation/reimbursement. There is no immediate need to make decision regarding implementation at this time.

Supplementary Text 3: organizational issues

Text 3: Details on the organizational issues considered in this health technology assessment

In this HTA, there were several organizational aspects considered important for possible implementation of the PREVENTOMICS interventions, which are summarized in the text below. They were divided based on the topics suggested in the HTA core model.

Health delivery process

Overall, the PREVENTOMICS interventions could be considered supplementary on the current work of health care professionals (i.e., nutritionists, dieticians, or other professionals). Nutritionists are likely very familiar with the use of apps to document health behaviors, and they will also be familiar with many of the tests performed to personalize nutrition. However, the specific tests required for personalized nutrition, might not be used in current practice. A few examples of responses from partners of the PREVENTOMICS project, who are experts in this field, related to the way the PREVETNOMICS interventions might influence their current work are given below.

One response from Spain regarding the effect was the following:

> *"[The technology] could be considered supplementary to the way it is currently applied by nutritionists. Nutritionists usually do not ask for a genetic test nor a metabolic analysis. However, we have proved that looking at the scores gives more insights on the metabolic status than trusting the anthropometrics alone. The goal would be to move towards this type of personalization in daily practice. Also, to be used as a stand-alone service (e.g., PREVENTOMICS as a service) you might ask for a genetic test and come for the analysis or make arrangements with laboratories (equipped accordingly) where the user can go to take the samples."*

A response from the UK was very similar:

> *"I see this as a supplement to nutritionist current practice, giving additional objective measurements that can be used to improve individual's understanding of the role of nutrition in health and the importance of making dietary change."*

A response from Denmark was also quite similar:

> *"The technology can supplement professional dieticians' current practice in a way that uses additional biomarkers to improve individuals' health outcomes. However, this will require professionals to be knowledgeable about the technology and the use of genetics in clinical practice."*

It will be important to verify whether the staff involved in providing personalized nutrition are able to perform the required tasks. Additionally, it might be important to educate and train the professionals in how to provide food recommendations, since this can lead to better results. One finding that supports this, relates to the pilots in the UK and Poland, that aimed to have similar design. However, results in the UK were more beneficial than in Poland, which could possibly be explained by the finding which is explained below:

> *"What I [UK researcher] found really interesting is that Controls had only standard recommendations, but for PP and PN, I got really involved when providing nutritional plans, explaining to volunteers the scientific basis underlying the foods that had been selected for them and even challenging them when asking for the reason to choose one or another food related with their metabolic cluster. I also elaborated more developed explanations for clusters and defined specific foods to increase/decrease for the different food categories coming out from the Nutrition Recommendation Engine. This "didactic" way of providing recommendations together with extended elaboration of food recommendations was the only point that was not standardized for both pilots [UK and Poland] and might be a plausible explanation for the differences."*

Overall, the PREVENTOMICS interventions did not require any new forms of co-operation and communication of activities. That is, the need to receive the lab results might require some change in co-operation. However,

coordination on partners analyzing different complementary aspects on the same sample requires good communication/collaboration. In contrast, some elements of personalized nutrition might require new activities related to quality assurance. For example, it would be important to monitor whether the cluster to which a participant is assigned is the correct one, and also to monitor whether the participant was later actually assigned to the correct cluster.

On the other hand, there are the participants. The participant's flow does not change much. However, one change in flow associated with the personalized nutrition is the need for additional testing before the participant would be allocated to a cluster. Currently, participants would receive dietary advice without any prior testing. This change currently results in a delay, which may decrease enthusiasm and perhaps compliance. In contrast, participants are required to use the app to document their food habits and other information. This requires additional instruction and can also require ways to keep the participant motivated to provide the information. Additionally, an aspect not raised by the participants but worth considering is the size of the household. In households with more than one member who dine together, the practicality of preparing individualized meals for each family member should be addressed. This poses a question about the feasibility of such an approach, especially for families or larger households.

Structure of health care system

(De)centralization issues are unlikely to have any influence on the implementation of personalized nutrition interventions. For example, the health professionals involved in personalized nutrition (including nutritionists) can be found in every health center.

Process-related costs

The process-related costs for the PREVENTOMICS interventions are expected to be low. No new hardware would need to be purchased. Also, the software costs to provide personalized nutrition interventions are relatively low. For example, the license to use the MetaDieta app has a price of 3000 euros per year. It would probably also not influence the need for other technologies, especially not in the short term. If it results in weight loss and improved health, it could perhaps reduce the need for treatment and hospitalization.

Management

In the pilots of the PREVENTOMICS project, there were some management problems and opportunities attached to the interventions. A nutritionist from the UK reported major problems regarding the delay in receiving results needed to personalize the intervention. Specifically, there was a delay of 2-3 months from receipt of samples to receiving the results. This can result in a loss of momentum and motivation by the individual. In addition, she noted that *"the material provided needs a lot of work to be more readily usable and valuable to the dietician. Currently the onus is on the dietician to develop a lot of the supporting material and food lists, which is a loss of value added that PREVENTOMICS could otherwise capture, and which competitors could easily seize [on] to gain competitive advantage."*

However, it must be noted that there were logistical issues due to the pandemic and therefore it was not possible to analyze [samples] in one go. Moreover, some samples needed to be taken again in the UK, as they were stopped at the border because of Brexit.

A response from Spain was as follows:

> *"Clear logistic pathway, arrangement with laboratories. For the project we have [split] analysis into different partners, ideally, and as a service, better to minimize this or centralize as much as possible. We have observed also the need for clear instructions to volunteers in case they need to take samples themselves (e.g., saliva)."*

The response from Poland was as follows:

> *"The nutritionists reported some minor shortcomings during the pilot, which were resolved on an ongoing basis (e.g., data flow between DSS and MetaDieta)."*

A response from Denmark was as follows:

"Many of our participants were highly motivated when recruited for the study, but the design of our pilot where that all participants need to start within limited time-period. This meant that the first participants were recruited in late October, had their first visit in January and did not start up on the actually diet before mid March. Some of the participants have later commented on this long waiting period even though they were told before they signed up for the study. Luckily, we only had 7 dropouts from when we stopped recruiting (mid Dec) to the first visits (mid Jan).

Furthermore, we experienced that the platform was down half of one day, meaning that all questionnaires were filled out in hand, and were entered to the platform by staff the day after.

Moreover, other small technical issues occurred during the visit day, where a few numbers of phones could not install the apps. This was, however, solved ad hoc."

In sum, different problems have been encountered, but have already been resolved.

Culture

The PREVENTOMICS interventions were overall well accepted by the participants. One response from Spain:

"[The system was] well accepted [by participants]. ...check this blog post prepared by OCU: https://preventomics.eu/requirements-for-a-e-health-tool-from-consumers-point-of-view/. This blog post describes the different principles (list of requirements) that were applied when developing the PREVENTOMICS e-health tool."

The response from the UK was somewhat less enthusiastic:

"[Study participants] engaged well with the results presented. However, the mobile app was not easy to use and needs substantial work. It currently only allows the participant to log food intake and compare this to the dietary prescription. It did not highlight foods to include according to cluster, provide recipes, assist with creating shopping lists with alternatives more aligned to the cluster, etc. There are a lot of better apps on the market (e.g., My Fitness Pal) which are far easier to use and offer greater functionality."

However, it must be said that the objective of this pilot was not the app itself, but the software for the professionals. The app was something that can be seen as "additional".

The response from Poland highlighted both strengths and weaknesses of the system and the app:

"Most of the volunteers emphasized that the great advantage of this project is the possibility of having results not only of routine tests, but also new ones, which are currently discussed in the media, e.g., genetic risk score and intestinal microbiota tests. Regarding the use of the mobile MetaDieta app for participants, generally individuals found it to be helpful in the dietary intervention. Unfortunately, they reported that it was not possible to select certain food products to be recorded in the app. Although volunteers were given suggestions of food substitutes in the dietary plans by their nutritionists (to be used interchangeably in a meal at different weeks), they could not see these substitutes in their mobile app. So, some improvements to the functionality of the application are desired."

A response from Denmark was as follows:

"Some of the Danish participants loved to browse the SF app while other did not use it much. The app where there for inspiration so for lunches and for the day they did not received food for. The OMNI app worked more or less without any bigger issues. For the Danish study, the platform was mainly used together with staff, but many found it confusing to navigate in."

Supplementary Text 4: legal aspects

Text 4: Details on the legal aspects considered in this health technology assessment

In this HTA, several legal aspects were considered important, which are summarized in the text below. They were divided based on the topics suggested in the HTA core model. Patient autonomy was handled in a different domain (see ethical aspects above) and is not discussed here.

Privacy of the patient

Under EU standards, personal data is defined as any information related to an identified or an identifiable person (art. 4.1 General Data Protection Regulation (GDPR)) (29). Anonymized data does not fall under this definition, but the bar for “anonymized data“ is set very high.

The processing of personal data (including health data and genetic data) includes (but is not limited to): collection / storage / structuring / adaptation / consultation / transmission / destruction (art. 4.2 GDPR). It is for any supplier of personalized nutrition of essence to only process personal data based on a valid legal basis, such as a consent (art. 6 GDPR). The definition of “Consent” is as follows: any freely given, specific, informed, and unambiguous indication of a data subject’s agreement with the processing of his/her personal data based on a statement or clear affirmative action (art. 4.11 GDPR). The use of consent as a legal basis for the processing of personal data is further detailed in Guidelines 05/2020 by the European Data Protection Board.

If there is a valid legal basis, it remains prohibited to process health, genetic and other sensitive data unless specific conditions have been met (article 9 GDPR). This is for instance the case when the ‘data subject’ has given its specific consent for processing for a specific purpose (art. 9.2 (a) GDPR), or when processing is necessary for scientific purposes (art. 9.2 (j) GDPR).

In the PREVENTOMICS project, the collected data was to be stored in a secure server, only visible to the research site network (Deliverable 6.1 (‘Ethical framework’)). Anonymous and identifiable data was to be stored separately, and only the project authorized person(s) could have access to the stored data. Anonymity was guaranteed by separating identifiable data from anonymous data. Anonymous data was to be made available to researchers. If any identifiable data was required for the research purposes, access, and distribution to it was to be granted only after explicit permission and after agreement of the data holders (participants providing the data). Authentication was required to access stored data on the research site.

Researchers handling and processing personal and sensitive data within the project were asked to sign a statement that they were familiar with and abided by the contractual obligations of the consortium. If not included in this obligation, they had to sign a statement that committed them to ensure project data were not provided to persons outside the project consortium.

Equality in health care

There is a variety of laws and binding rules that guarantee equal access to technologies, in which there are also cross-country differences. One example of a European wide right, that ensures equal access, is the non-discrimination right (30). This law prohibits discrimination based on factors such as race, sex, age, disability, or socioeconomic status. By means of this law, everyone should have an equal opportunity to access and benefit from personalized nutrition regardless of their personal characteristics or circumstances. Another example is the general food law (31), that states that European citizens need to have access to safe and wholesome food of highest standards.

However, there are cross-country differences in rules and regulations regarding equality in health care. For example, as mentioned before in Supplementary Text 1 and domain ‘description and technical characteristics of the technology’, there are cross-country differences in reimbursement and insurance coverage. Some countries have health insurance systems that cover digital health or specific medical services, including nutritional counseling or consultations, while others have not. The availability and extent of insurance coverage for personalized nutrition services can significantly impact accessibility, as those without coverage may face financial barriers to accessing such services (see also ‘ethical’ domain).

Ethical challenges of existing legislations

Testing involved in personalizing nutrition looks like tests that are currently available and it is therefore unlikely that use of the technology will lead to ethical challenges that have never been considered before and not addressed in existing legislation. However, one of the main objectives of current legislation is privacy and data protection. The kinds of analyses in PREVENTOMICS, mainly genetics, but also metabolomics, could provide large volumes of information about the user that might put anonymization at risk. Therefore, care must be taken when this data is used and shared with others. Moreover, it must be noted that personalized nutrition is a multifaced phenomenon, so many different rules and regulations need to be combined, in which blurred boundaries exist between “health” and “lifestyle” products and “food” or “medicine” (32).

Authorization and safety

The MetaDieta app/software should be considered as a “medical device”. A medical device is any device, software or other article intended by the manufacturer to be used for specific medical purposes (e.g., prevention of a disease) (29). Therefore, the Medical Devices Regulation (MDR) is applicable in which a CE mark was needed (33).

Ownership and liability

Deliverable 7.5 (‘Final plan for the Use and Dissemination of Results-PUDR’) provides information on the dissemination and exploitable results of the PREVENTOMICS project, including ownership rights and intended IPR protection strategies, as well as a summary of dissemination actions and future activities. In brief, two different approaches were suggested, which allow for flexibility of choice among partners. This is necessary since this is a complex project and the management of multiple relationships between partners and a new approach was set up within the Joint Exploitation Routes. These approaches are: Joint Venture and Licensed results.

First, regarding a joint venture approach, the vision will be to create a separate company or legal entity where ownership is distributed based on partners’ allocated efforts and contribution to each of the project developments. The Joint Venture will have its own structure: its shareholders are expected to be the core partners of the project plus any external 3rd party company that provided added value and wishes to join the venture. The Joint Venture will have its own team of dedicated professionals, such as technicians, managers, engineers and commercial agents. An IP Entity Manager can be appointed to deal with business development matters as well as neutral administrative work.

The second approach is a licensed results approach, which is based on licensing the results of the project into separate entities that will deal with the commercialization of the PREVENTOMICS platform. This way, there are no direct legal relationships amongst the project partners, which can facilitate and speed the go-to-market strategy of the technologies, as there are no complicated negotiations. Two options in this approach are available for exploration: IP Brokering and IP Transfer. Both of these are set on the philosophy that the project results are licensed to an intermediary, which will receive a percentage fee for the commercialization efforts and can include the PREVENTOMICS Platform in their own portfolio of services/products and their business model.

Regulation of the market

In the case of personalized nutrition, it is unlikely that there are any relevant price control mechanisms. However, further assessment is needed to verify this. Moreover, it is unlikely that there are any legal restrictions to marketing the personalized nutrition. This is partly because the target population does not include persons with serious diseases and also because the forms of personalized nutrition developed in PREVENTOMICS do not involve any important health risks. It is however important for developers of any personalized nutrition intervention to consider whether or not their product will be seen as a medical device. This is not easy to determine since it remains the question when a device will transform from a lifestyle product to a medical device. For example, an app with diet recommendations based on potential health data can be seen as a lifestyle product. However, when its developers claim that the app can help to address or threat a medical condition like obesity, it transforms into a medical device. Additionally, developers of

personalized nutrition interventions also need to be aware that the food market is highly regulated by the European Food Safety Authority (EFSA) (see Deliverable 7.4 (‘PUDR’)) (32).

References

1. Centers for disease control and prevention. Adult Body Mass Index [Internet]. 2021 [cited 2022 Apr 20]. Available from: https://www.cdc.gov/obesity/basics/adult-defining.html?CDC_AA_refVal=https%3A%2F%2Fwww.cdc.gov%2Fobesity%2Fadult%2Fdefining.html

2. Bothos E. PREVENTOMICS Final Conference: Recommender system, integration in third-party apps [Internet]. 2022 [cited 2023 Jan 5]. Available from: https://preventomics.eu/preventomics-final-conference/

3. Poley MJ. Nutrition and health technology assessment: when two worlds meet. Front Pharmacol. 2015;6(232):1–6.

4. EUnetHTA WP7. An analysis of HTA and reimbursement procedures in EUnetHTA partner countries: final report. [Internet]. Eunethta. 2017. Available from: http://eunethta.eu/outputs/analysis-hta-and-reimbursement-procedures-eunethta-partner-countries-final-report

5. Perugini M, Johnson TJ, Beume TM, Dong OM, Guerino J, Hu H, et al. Are We Ready for a New Approach to Comparing Coverage and Reimbursement Policies for Medical Nutrition in Key Markets: An ISPOR Special Interest Group Report. Value in Health. 2022;25(5):677–84.

6. Bush CL, Blumberg JB, El-Sohemy A, Minich DM, Ordovás JM, Reed DG, et al. Toward the Definition of Personalized Nutrition: A Proposal by The American Nutrition Association. J Am Coll Nutr. 2020;39(1):5–15.

7. Laegemiddelstyrelsen: Danish Medicines Agency. Application for reimbursement of nutritional products from the Regional Council [Internet]. 2023 [cited 2023 Mar 31]. Available from: https://laegemiddelstyrelsen.dk/en/reimbursement/reimbursable-nutritional-products/application-for-reimbursement/

8. Briggs Early K, Stanley K. Position of the Academy of Nutrition and Dietetics: The Role of Medical Nutrition Therapy and Registered Dietitian Nutritionists in the Prevention and Treatment of Prediabetes and Type 2 Diabetes. J Acad Nutr Diet. 2018;118(2):343–53.

9. Kostelecki G, Mastalerz-Migas A. Guidelines of the Polish Society of Dietetics and a national consultant in the field of family medicine regarding dietary consultations as part of coordinated care in primary health care of January 31, 2023 [Internet]. 2023. Available from: https://www.gov.pl/attachment/2968553a-2aef-4d31-9ec3-c34a9d7844c7

10. Minister of Health. Regulation of the Minister of Health (Rozporządzenie Ministra Zdrowia z dnia) [Internet]. Journal of Laws. 2022. Available from: https://dziennikustaw.gov.pl/ D2021000064201.pdf

11. Brotonsc C, Björkelund C, Bulc M, Ciurana R, Godycki-Cwirko M, Jurgova E, et al. Prevention and health promotion in clinical practice: The views of general practitioners in Europe. Prev Med. 2005;40(5):595–601.

12. Uerlich MF, Yumuk V, Finer N, Basdevant A, Visscher TLS. Obesity management in Europe: Current status and objectives for the future. Obes Facts. 2016;9(4):273–83.

13. Freeman KJ, Grega ML, Friedman SM, Patel PM, Stout RW, Campbell TM, et al. Lifestyle medicine reimbursement: A proposal for policy priorities informed by a cross-sectional survey of lifestyle medicine practitioners. Int J Environ Res Public Health. 2021;18(21):1–16.

14. Zwaagstra Salvado E, van Elten HJ, van Raaij EM. The Linkages Between Reimbursement and Prevention: A Mixed-Methods Approach. Front Public Health. 2021;9(750122).

15. OECD. Healthy Eating and Active Lifestyles: Best Practices in Public Health: 5. Combined Lifestyle Intervention [Internet]. Paris; 2022 [cited 2023 Mar 31]. Available from: https://www.oecd-

ilibrary.org/sites/51d0a464-en/index.html?itemId=/content/component/51d0a464-en#section-d1e22821

16. Brinkmann-Sass C, Richter L, Silberzahn T, Somauroo A. McKinsey & Company. 2020 [cited 2023 Mar 31]. The European path to reimbursement for digital health solutions [Internet]. Available from: https://www.mckinsey.com/industries/life-sciences/our-insights/the-european-path-to-reimbursement-for-digital-health-solutions

17. MedTech Europe. Recognising the value of digital health apps : An assessment of five European healthcare systems [Internet]. 2021. Available from: https://www.medtecheurope.org/resource-library/recognising-the-value-of-digital-health-apps-an-assessment-of-five-european-healthcare-systems/

18. Lorenzo D, Mirza M, Wu Y. 2 Years After Germany's Regulation For Digital Health Apps, What Can We Learn? [Internet]. 2022 [cited 2023 Jun 2]. Available from: https://www.meddeviceonline.com/doc/years-after-germany-s-regulation-for-digital-health-apps-what-can-we-learn-0001

19. Gerke S, Stern AD, Minssen T. Germany's digital health reforms in the COVID-19 era: lessons and opportunities for other countries. Digit Med. 2020;3(1):1–6.

20. Bundesinstitut fur Arzneimittel und Medizinprodukte. Oviva Direct for obesity [Internet]. 2023 [cited 2023 Jun 2]. Available from: https://diga.bfarm.de/de/verzeichnis/00872

21. Ahlgren J, Nordgren A, Perrudin M, Ronteltap A, Savigny J, Van Trijp H, et al. Consumers on the Internet: Ethical and legal aspects of commercialization of personalized nutrition. Genes Nutr. 2013;8(4):349–55.

22. Castle D. The ethics of personalized nutrition. Agro Food Ind Hi Tech. 2007;18(2):68–70.

23. Korthals M. Prevention of Obesity and Personalized Nutrition: Public and Private Health. In: Genomics, Obesity and the Struggle over Responsibilities. Springer Science+Business Media B.V.; 2011. p. 191–205.

24. Aldubayan MA, Pigsborg K, Gormsen SMO, Serra F, Palou M, Mena P, et al. Empowering consumers to PREVENT diet-related diseases through OMICS sciences (PREVENTOMICS): protocol for a parallel double-blinded randomised intervention trial to investigate biomarker-based nutrition plans for weight loss. BMJ Open. 2022;12(3):e051285.

25. Del Bas JM. NCT04641559: Personalized Nutrition Advice for Optimizing Dietary Habits and Metabolic Status (PREVENTOMICS) [Internet]. 2022 [cited 2023 Jan 6]. Available from: https://clinicaltrials.gov/ct2/show/NCT04641559?term=preventomics&draw=2&rank=1

26. Calder P. ISRCTN51509551: Personalised advice to aid weight loss [Internet]. 2021 [cited 2023 May 17]. Available from: https://www.isrctn.com/ISRCTN51509551?q=personalised nutrition advice&filters=&sort=&offset=9&totalResults=22&page=1&pageSize=10

27. Malczewska-Malec M. ISRCTN46063864: Personalised nutritional advice to aid weight loss [Internet]. 2022 [cited 2023 May 17]. Available from: https://www.isrctn.com/ISRCTN46063864?q=preventomics&filters=&sort=&offset=1&totalResults=1&page=1&pageSize=10

28. Mathers JC. Paving the way to better population health through personalised nutrition. EFSA Journal. 2019;17(S1):1–9.

29. Verzijden K, Buijs J, de Boer A, Rottger-Wirtz S. Food Health legal (legal and regulatory blog) [Internet]. 2021 [cited 2022 Apr 26]. The regulatory framework of personalised nutrition. Available from: http://foodhealthlegal.eu/?p=1108

30. European Commission. Non-discrimination [Internet]. 2023 [cited 2023 Jul 14]. Available from: https://commission.europa.eu/aid-development-cooperation-fundamental-rights/your-rights-eu/know-your-rights/equality/non-discrimination_en

31. European Commission. General Food Law [Internet]. 2023 [cited 2023 Jul 13]. Available from: https://food.ec.europa.eu/horizontal-topics/general-food-law_enequity or eq

32. Röttger-Wirtz S, De Boer A. Personalised Nutrition: The EU's Fragmented Legal Landscape and the Overlooked Implications of EU Food Law. European Journal of Risk Regulation. 2021;12(1):212–35.

33. European Commission. CE marking [Internet]. [cited 2023 Apr 4]. Available from: https://single-market-economy.ec.europa.eu/single-market/ce-marking_en
